# Nup107 contributes to the maternal-to-zygotic transition by preventing the premature nuclear export of pri-miR427

**DOI:** 10.1242/dev.202865

**Published:** 2025-02-04

**Authors:** Valentyna Kostiuk, Rakib Kabir, Kaitlin Levangie, Stefany Empke, Kimberly Morgan, Nick D. L. Owens, C. Patrick Lusk, Mustafa K. Khokha

**Affiliations:** ^1^ Pediatric Genomics Discovery Program, Departments of Pediatrics and Genetics, Yale School of Medicine, 333 Cedar Street, New Haven, CT 06520, USA; ^2^ Department of Cell Biology, Yale School of Medicine, 295 Congress Avenue, New Haven, CT 06520, USA; ^3^ Department of Clinical and Biomedical Sciences, University of Exeter, Barrack Road, Exeter EX2 5DW, UK

**Keywords:** Nucleoporins, miR427, Maternal-to-zygotic transition, microRNA, Germ layers patterning, Nuclear transport, *Xenopus*

## Abstract

Emerging evidence suggests that the nuclear pore complex can have unique compositions and distinct nucleoporin functions in different cells. Here, we show that Nup107, a key component of the NPC scaffold, varies in expression over development: it is expressed at higher levels in the blastula compared to the gastrula, suggesting a crucial role before gastrulation in *Xenopus*. We find that depletion of Nup107 affects the differentiation of the early germ layers leading to an expansion of the ectoderm at the expense of endoderm and mesoderm. By analyzing an RNA-sequencing time course, we observed that depletion of Nup107 affects the maternal-zygotic transition by delaying the degradation of maternal transcripts that occurs as zygotic transcription begins. The transcripts are enriched in recognition sites for miR427, a conserved microRNA that destabilizes maternal transcripts including REST, which encodes a Kruppel-type zinc-finger transcription factor that we demonstrate is crucial for ectodermal cell fates. Mechanistically, we show that Nup107 is required to prevent the premature export of *pri-miR427* transcript before processing. Nup107 depletion leads to the reduced production of mature miR427 and maternal transcript stabilization. We conclude that high levels of Nup107 in the early embryo are crucial for the nuclear retention and subsequent processing of *pri-miR427* transcripts that is required for timely maternal RNA clearance to enable gastrulation.

## Teaser

**Highlighted Article:** This study explains the role of Nup107 in maternal-to-zygotic transition through primary miR427 nuclear export.

## INTRODUCTION

Nucleoporins (nups) are components of nuclear pore complexes (NPC), transport channels embedded in the nuclear envelope that control molecular exchange between the cytoplasm and nucleus. Although NPCs are generally thought to be biochemically and thus functionally identical in most cell types, there is emerging evidence of cell type-specific expression of nup genes that might contribute to cell fate decisions (Cho and Hetzer, 2020; D'Angelo et al., 2012; Reza et al., 2016). The underlying mechanisms by which cell type-specific changes in nup levels impact differentiation pathways remains ill-defined but may be relevant to understanding why nups also contribute to a wide array of tissue-specific diseases (Muir et al., 2020; Braun et al., 2016; Park et al., 2020, 2017, 2014; Miyake et al., 2015; Weinberg-Shukron et al., 2015; del Viso et al., 2016).

An individual NPC is composed of ∼30 nups that come together in multiple copies to form the eightfold radially symmetric, 100 MDa macromolecular assembly that is embedded in the nuclear envelope (Beck and Hurt, 2017). The central channel of the NPC contains the Phe-Gly-rich (FG) nups that form a size-selective diffusion barrier and interact with nuclear transport receptors carrying cargoes (Cautain et al., 2015; Shen et al., 2021). The FG nups are anchored to the NPC scaffold, which consists of three concentric ring assemblies: the cytoplasmic ring, the inner ring, and the nucleoplasmic ring. The major nup components of the cytoplasmic and nucleoplasmic rings are the Nup107-160 ‘Y-complex’.

Although multiple components of the Y-complex have been linked to disease, *NUP107* stands out as heterozygous loss-of-function variants in *NUP107* were identified in two individuals with left ventricular outflow tract obstruction, indicating a malformation in the left heart chambers (Jin et al., 2017). Besides cardiac abnormalities, *NUP107* variants have been reported in individuals with steroid-resistant nephrotic syndrome, microcephaly, hypergonadotrophic hypogonadism, gonadal dysgenesis and infertility (Rosti et al., 2017; Park et al., 2020, 2017; Miyake et al., 2015; Ren et al., 2018; Braun et al., 2018; Weinberg-Shukron et al., 2015). This broad spectrum of birth defects suggests that Nup107 could be involved in several independent pathways, or it could affect an early embryonic patterning process leading to subsequent defects in multiple embryonic structures. To investigate how Nup107 impacts embryonic development, we took advantage of the rapid external development of *Xenopus* embryos. We show that Nup107 facilitates the maternal-to-zygotic transition by preventing the premature nuclear exit of *pri-miR427* transcripts. In turn, this contributes to the clearance of maternal transcripts allowing proper germ layer patterning and organogenesis. Thus, our work provides evidence for how nups, present in all cell types, can impact stage-specific developmental transitions.

## RESULTS

**Fig. 1.**
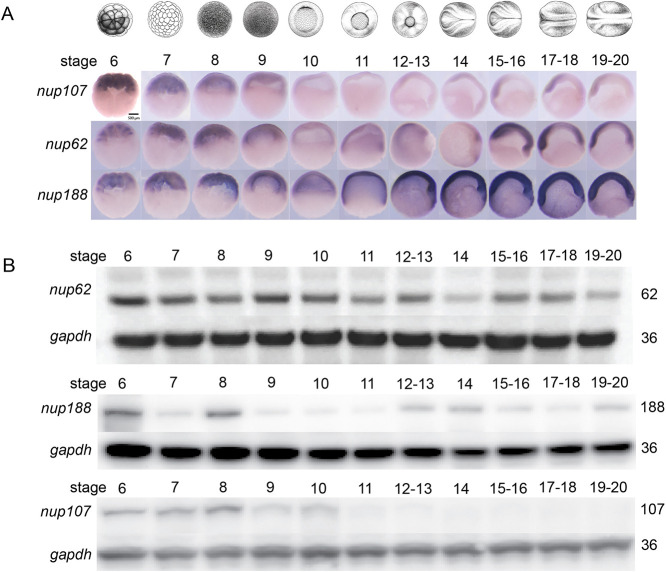
**Spatio-temporal patterns of nucleoporin expression.** (A) mRNA expression domains of *nup107*, *nup62*, and *nup188* detected by whole-mount *in situ* hybridization using antisense probes at different developmental stages (panel above). Scale bar: 500 μm. (B) Protein expression levels of Nup62, Nup188, and Nup107 detected by western blotting at different developmental stages (panel above). *Gapdh* is a loading control. Three biological replicates were used for all experiments, with 30 embryos used per stage per condition. All data represent results from experiments replicated at least three times in the laboratory.

Given that Nup107 variants are associated with multiple tissue-specific diseases, we first examined its spatio-temporal expression by whole-mount *in situ* hybridization at different developmental stages from stage 6 (32 cell) to stage 20, when organogenesis begins (Fig. 1A). We compared the Nup107 expression pattern to that of Nup62 (an FG nup) and Nup188, which encodes for a scaffold/inner ring nup, which we have previously shown plays a role at cilia (Fig. 1A) (del Viso et al., 2016; Vishnoi et al., 2020; Marquez et al., 2021). The signal from the *nup107* transcripts was higher during early development and lower after the onset of gastrulation (stage 10). This expression pattern was unique to *nup107*, as *nup188* mRNA was also detected during gastrulation (stages 10-12) and later in the neural folds of embryos undergoing neurulation (stages 13-20). Interestingly, the *nup107* RNA expression detected by RNA-seq using polyA+ and ribozero samples also showed high transcript levels before gastrulation, with a significant decrease right at the beginning of gastrulation (stage 10) (Fig. S2A,B). When we examined protein levels by western blot, we also noticed variability across development, especially for Nup188 and Nup107*.* The protein levels of Nup188 had a wave-like character that peaked at early (stage 8) and at later (stages 12-14) stages. Interestingly, stages 12-13 are associated with cilia formation and function. Surprisingly, we detected high levels of Nup107 protein before stage 10, which marks the onset of gastrulation. In comparison, the expression of Nup107 protein was diminished at later stages (Fig. 1B). Thus, nup levels vary considerably relative to each other across development.

**Fig. 2.**
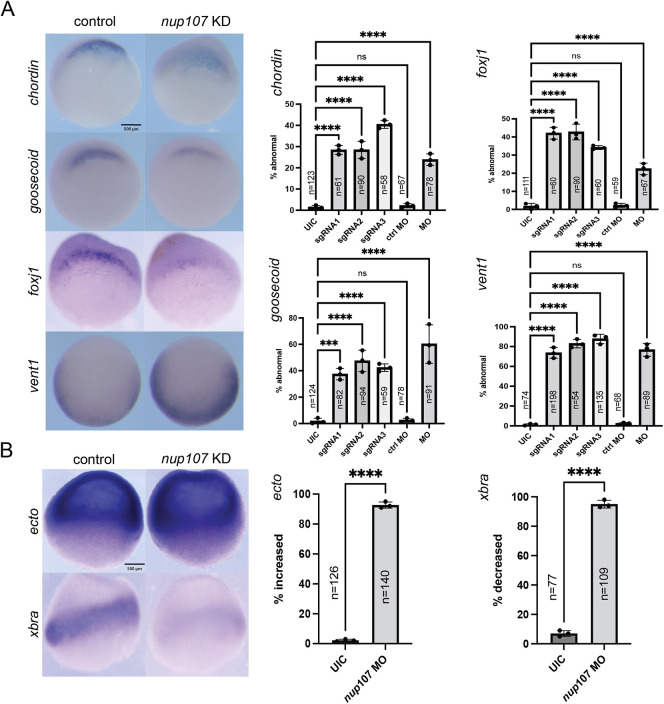
**Nup107 depletion alters germ layer specification.** (A) Representative images of whole-mount *in situ* hybridization embryos at stage 10 demonstrating reduced expression levels of dorsal mesodermal markers (*chordin*, *goosecoid*, *foxj1*) and upregulated expression of a ventral mesodermal marker (*vent1*) due to Nup107 depletion. (B) Whole-mount *in situ* hybridization images of embryos at stage 10 showing upregulated ectodermal marker (*ectodermin*) and reduced mesodermal marker (*xbra*) expression in Nup107*-*depleted embryos. Three biological replicates were used for all experiments. All data represent results from experiments replicated at least three times in the laboratory. Statistical significance was determined using two-way ANOVA analysis. ***P<0.001, ****P<0.0001. ns, not significant. Data are mean±s.d. Scale bars: 500 μm.

Surprised by the finding that Nup107 protein levels are diminished before gastrulation, we investigated a role for Nup107 at this timepoint. We used CRISPR/Cas9 to introduce indels into the *nup107* gene to ablate its expression. We found that 36±12.8% (mean±s.d.) of embryos depleted of Nup107 did not gastrulate normally (compared to 0% in controls) (Fig. S3). For gastrulation to progress, two processes are essential: germ layer patterning and morphogenesis (Huang and Winklbauer, 2018; Solnica-Krezel and Sepich, 2012; Heisenberg and Solnica-Krezel, 2008). Immediately before gastrulation, the blastula embryo is divided into three germ layers: the ectoderm, mesoderm, and endoderm. The mesoderm initiates gastrulation on the dorsal side of the blastula at a crucial signaling site known as the Spemann-Mangold Organizer (Organizer) (Spemann and Mangold, 1923). Markers of the Organizer (Fig. 2A) were reduced in Nup107-depleted embryos. Approximately 28% and 45% of Nup107-depleted embryos had decreased expression of *chordin* and *goosecoid*, respectively. Similarly, *foxj1* levels were lower in approximately 35% of Nup107-depleted embryos (compared to 2% in controls). The reduction of dorsal mesodermal markers could be due to expansion of ventral mesodermal markers. To assay ventral mesodermal fates, we examined the expression of *vent1*. More than 70% of Nup107-depleted embryos had increased expression of *vent1* (compared to 2% in controls); however, its expression domain did not expand into the dorsal region (Fig. 2A).

To test the specificity of our depletion strategies, we employed three different sgRNAs, all of which generated similar phenotypes in F0 CRISPR embryos (Fig. 2; Fig. S4). Additionally, we confirmed CRISPR-Cas9 cutting efficiency using inference of CRISPR edits (ICE) analysis for all three sgRNAs (Fig. S4). We also depleted Nup107 with a start site morpholino oligonucleotide (MO), which produced a similar phenotype as well (Fig. 2). To test the specificity of the MO, we demonstrated rescue with human *NUP107* mRNA in Nup107 MO-depleted embryos (Fig. S5A). Finally, to test the efficiency of our MO and specificity of the antibody, we detected a reduction in Nup107 protein by western blot in MO-depleted embryos and an increase in Nup107 protein with human mRNA overexpression (Fig. S5B). Therefore, based on these tests, we conclude that the phenotypes associated with Nup107 depletion are specific to the loss of function of Nup107.

Since the expression of both the dorsal and ventral mesoderm was abnormal under conditions of Nup107 depletion, we tested the expression of a pan-mesodermal marker *xbra* (*tbxt*), which was reduced in 93% of Nup107-depleted embryos (and 7% in controls) (Fig. 2B). Given the loss of general mesodermal cell fates, we next examined the other two germ layers: ectoderm and endoderm. Similar to mesoderm, endodermal markers, *vegT* and *mixer*, were reduced in the majority of Nup107-depleted embryos (84% and 97%, respectively, compared to 16% and 2% in controls) (Fig. S6). However, the ectoderm, marked with *ectodermin* and *foxi1a* (*foxi3.2*), was expanded into the mesendodermal domain in over 80% of Nup107-depleted embryos (compared to 2% in controls) (Fig. 2B; Fig. S6). Additionally, we wondered whether the Nup107 depletion phenotype would be mirrored by the depletion of other Nup107-160 complex members. Our results suggest that the depletion of Nup133 and Nup160 is distinct from Nup107 (Fig. S6). From these studies, we conclude that depletion of Nup107 leads to an alteration in germ layer fates whereby ectodermal fates are expanded at the expense of mesendodermal, which will affect gastrulation and embryonic patterning.

**Fig. 3.**
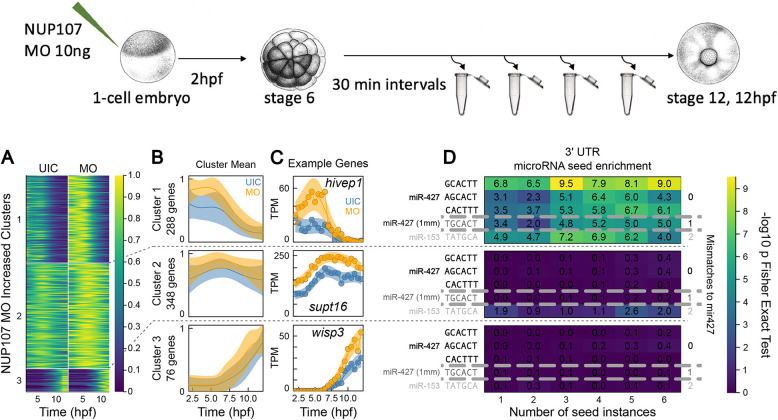
**Depletion of Nup107 alters temporal differential expression of specific genes.** (A,B) Summary of gene clusters with increased expression by heatmap (A) and cluster average (B). Clustered data is maximum normalized Gaussian process median expression for each gene. B provides mean±s.d. for each cluster. Decreased genes and ribozero clusters given in Fig. S7A,B. (C) Gene expression examples for each increased expression cluster for UIC and MO. Data points are gene expression in TPM; central line and shaded region are transformed Gaussian process median and 95% CI, respectively. (D) Enrichment of exact instances of microRNA seeds within 3′ UTRs of genes of each cluster. Color gives Fisher’s exact test −log10 *P*-value between the given number of instances and a gene being present in a cluster. Seeds organized by number of mismatches to miR427 sequences; see Materials and Methods for details. Schematic at top shows illustrates the experimental workflow. MO, *nup107* MO; UIC, uninjected control.

To investigate the relationship between Nup107 and germ layer patterning, we collected Nup107-depleted embryos and sibling controls every 30 min from early cleavage stages until the end of gastrulation (Fig. 3) and performed polyA+ RNA-seq, generating a time series of excellent quality (see Materials and Methods). Following a methodology we have used previously (Owens et al., 2016; Sempou et al., 2022), we identified genes with differential temporal expression, i.e. those genes for which expression trajectories differ between *nup107* MO and control embryos. We found that there were on average 712 genes activated and 247 genes repressed (Fig. S1C,D). We further subdivided these by k-means clustering into six clusters, three with increased expression U1-3 and three with decreased expression D1-3 (Fig. 3A; Fig. S7A). Broadly, for both increased and decreased expression classes, clusters 1-3 contain genes with differential expression from early to late in our time course.

We focused our attention on Cluster U1 (Fig. 3B,C, top panel) as this showed the earliest and largest divergences in gene expression and was coincident with the elevated expression of Nup107 that we observed by western blot (Fig. 1B). This cluster is composed entirely of maternal transcripts that show elevated expression at 2 h postfertilization (hpf) before widespread zygotic transcription (Owens et al., 2016). When assayed by polyA+ RNA-seq, maternal transcript levels will vary due to polyadenylation changes during early development (Owens et al., 2016; Collart et al., 2014). To distinguish whether the changes in cluster U1 were due to varying control of the polyA-tail or a variation in transcript levels, we repeated our RNA-seq time-course using a ribosomal RNA depletion (ribozero) protocol and differential expression analysis (Fig. S1A-E). Overall, we found excellent agreement between polyA+ and ribozero clusters of differentially expressed genes (Fig. S7A-D). Particularly, cluster U1 was highly concordant between polyA+ and ribozero sequencing (228/288 U1 polyA+ genes also in cluster U1 ribozero), demonstrating that the increases observed in U1 are due to changes in transcript levels rather than control of the polyA-tail.

The elevated transcript levels in cluster U1 appear at first surprising, as they occur before widespread zygotic transcription. They may result from failure to clear maternal transcripts, which can disrupt the maternal-zygotic transition leading to multiple developmental abnormalities, including germ layer mispatterning and gastrulation failure (Liu et al., 2020). There are multiple factors and mechanisms involved in clearing of maternal transcripts including AU-rich elements (ARE) (Voeltz and Steitz, 1998), EDEN and EDEN-specific RNA-binding protein (Paillard et al., 1998), miR430/miR427 (Lund et al., 2009), and the recognition of m^6^A modification by m^6^A reader protein YTHDF2 (Zhao et al., 2017). Among the mechanisms that clear maternal RNAs, *miR427* (the *Xenopus* ortholog of *miR430* in zebrafish) plays a prominent role in promoting the degradation of maternal transcripts in zebrafish and frog (Giraldez et al., 2006; Lund et al., 2009). To evaluate this possibility, we calculated the enrichment of all recognition motifs for ARE, EDEN, and YTHDF2 along with all microRNA seed sequences from miRbase (Kozomara et al., 2019) within the 3′ untranslated regions (UTRs) of genes in each cluster. We found that exclusively early activated clusters U1 and U2 were enriched in multiple microRNA seed sequences, whether evaluated by polyA+ or ribozero sequencing (Fig. S7E,F). Moreover, we found that among all miRbase seed sequences found in the 3′ UTR of genes in any cluster, sequences matching *miR427* were most strongly over-represented (Fig. S7E). When we examined the enrichment of multiple instances of miRbase sequences in any 3′ UTR of a single transcript, we found that the enrichment was exclusively for *miR427*, showing maximal enrichment at ≥6 seeds per UTR with both canonical and offset 6-mer sequences (Fig. 3D).

**Fig. 4.**
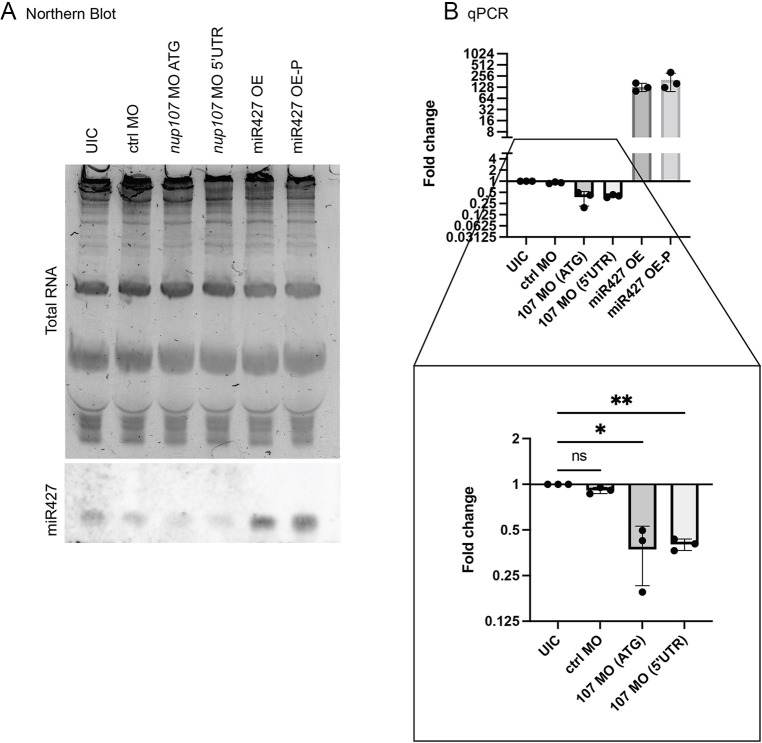
**miR427 expression levels at stage 8.** (A) Northern blot showing relative levels of miR427 in comparison to total RNA amounts; miR427 OE and miR427 OE-P are positive overexpression controls. (B) qPCR quantification of *miR427* expression levels using LNA probes for *miR427* and *u6* (small RNA standardization control). Three biological replicates were used for all experiments. All data represent results from experiments replicated at least three times in the laboratory. Statistical significance was determined using two-way ANOVA analysis. *P<0.05, **P<0.01. ns, not significant. Data are mean±s.d.

Given the enrichment of *miR427* seed sequences in Cluster U1, we visualized *miR427* by northern blot. We detected lower levels of *miR427* in Nup107-depleted embryos as compared to uninjected controls (UICs) and embryos injected with a control MO (Fig. 4A). To determine the specificity of our probe, we used two positive controls. We overexpressed the mature *miR427* by injecting the *miRNA* duplex (*miR427* OE). We also injected a modified *miR427* duplex with a 5′ phosphate moiety (*miR427* OE-P), which facilitates its RNA degradation activity and miRISC loading (Salzman et al., 2016; Medley et al., 2021). On northern blot, we detected an increased signal for *mir427* in embryos injected with these positive controls, indicating that our probe is specifically detecting *miR427* (Fig. 4A). We also used qRT-PCR to quantify *miR427* levels, which detected lower levels of *miR427* in Nup107-depleted embryos compared to control embryos (Fig. 4B). Embryos injected with either the *miR427* duplex or the phosphate modified duplex had an increase in qRT-PCR signal, again reflecting the specificity of our assay.

**Fig. 5.**
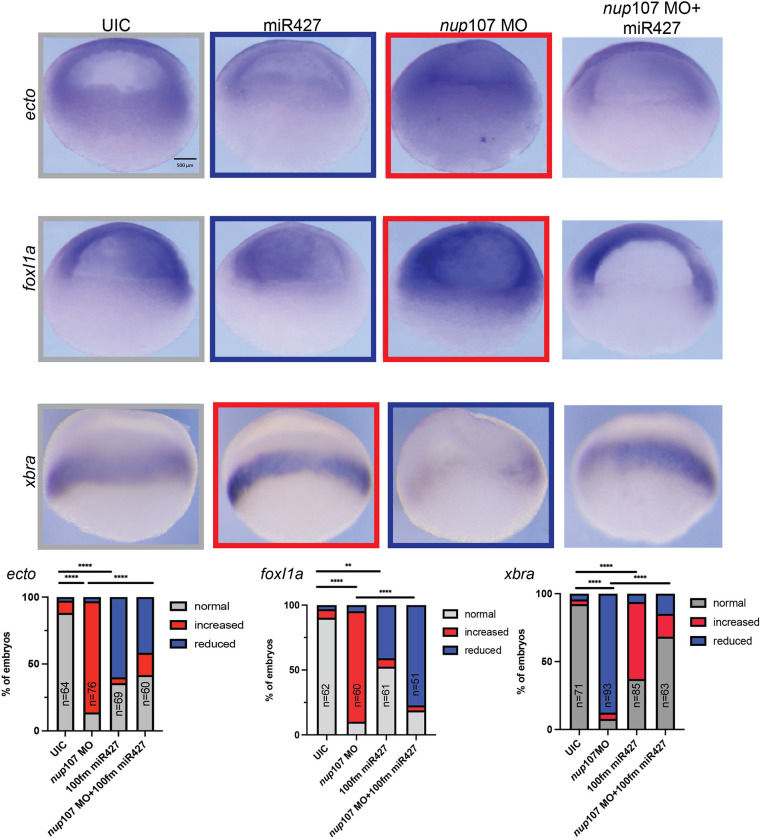
**miR427 overexpression rescues germ layer expression patterns associated with *nup107* depletion.** Increased ectodermal marker (*ectodermin* and *foxi1a*) expression due to Nup107 depletion (*nup107* MO) was rescued by *miR427* overexpression (*nup107* MO+miR427). The overexpression of *miR427* also rescued the reduced mesodermal signal (*xbra*) in Nup107-depleted embryos. Three biological replicates were used for all experiments. All data represent results from experiments replicated at least three times in the laboratory. Statistical significance was determined using two-way ANOVA analysis. **P<0.01, ****P<0.0001. ns, not significant. Scale bar: 500 μm.

Our results indicate that Nup107 depletion leads to a loss of *miR427*, which is essential for the clearance of maternal RNAs once zygotic transcription starts. Therefore, we predicted that exogenous administration of *miR427* should rescue the Nup107 depletion phenotype. To assay the Nup107 depletion phenotype, we evaluated the expression of ectodermal (*ectodermin* and *foxi1a*) and mesodermal (*xbra*) markers. Depletion of Nup107 leads to an increased expression of the ectodermal markers *ectodermin* and *foxi1a* (83% and 85% of embryos, respectively, compared to 9% and 7% in controls, respectively) (Fig. 5). However, injection of the duplex *miR427* caused a reduction in ectodermal marker expression (60% and 41% of embryos showing decreased *ectodermin* and *foxi1a* signals, respectively, compared to 3% in controls) (Fig. 5). Moreover, while we detected reduced expression of *xbra* in 88% of Nup107-depleted embryos (compared to 4% in controls), this phenotype was rescued by the overexpression of *miR427*, with only 15% of Nup107*-*depleted embryos showing reduced expression (Fig. 5). Therefore, addition of exogenous *miR427* rescued ectodermal and mesodermal patterning seen under conditions of Nup107 depletion. Additionally, we investigated whether the overexpression of *miR427* had an impact on events later in embryonic development by examining the expression of *pitx2c* (*pitx2*), a marker of left-right (LR) patterning. While 28% of Nup107*-*depleted embryos had abnormal *pitx2c* expression, concurrent overexpression of *miR427* reduced the occurrence of abnormal *pitx2c* to 11% of embryos (Fig. S8). This suggests that, in addition to its crucial role in maternal RNA clearance during early development, abnormal levels of *miR427* affect multiple downstream events, including LR patterning. Next, we sought to identify direct targets, as none of these marker gene transcripts (*ectodermin*, *foxi1a* or *xbra*) has *miR427* target sequences in their UTRs.

We previously showed that U1 is enriched for transcripts with *miR427* sites (Fig. 3D). We hypothesized that transcription factor transcripts stabilized by Nup107 loss may be responsible for activating genes dysregulated in other downstream clusters. Consistent with this idea, there were seven transcription factors in Cluster U1 for which we could identify their cognate binding motifs (see Materials and Methods). We assessed their enrichment in the promoters of genes within each of the three increased and three decreased clusters (Fig. S7G). Notably, we found *hivep1* motifs enriched in zygotic response promoters in U3 and D3, whereas *rest*, *arid4b*, and *znf518a* motifs were enriched across the promoters of all differentially expressed genes (Fig. S7G).

*Rest* emerged as an interesting candidate as it regulates ectodermal fates by defining the transition into neural cell fates instead of epidermal cell lineages. *Rest* plays a role in neuronal differentiation in zebrafish, frog and mouse embryonic stem cells (Wang et al., 2012b; Singh et al., 2008; Gupta et al., 2009; Olguin et al., 2006). In *Xenopus*, decreased *rest* expression leads to defects in the development of neural tube, cranial ganglia and eyes (Olguin et al., 2006). However, while *rest* appears to affect neuronal differentiation, a role in ectodermal germ layer patterning has not been defined.

**Fig. 6.**
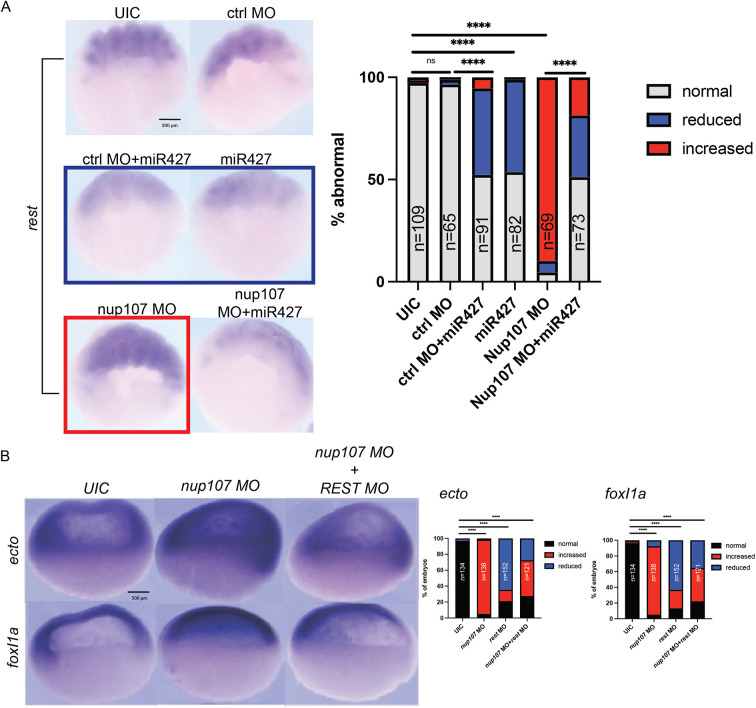
***rest* is a potential target of miR427 and a regulator of ectodermal patterning.** (A) Whole-mount *in situ* embryos at stage 8 demonstrating the effect of Nup107 depletion and *miR427* overexpression on the changes in *rest* expression patterns. (B) Whole-mount *in situ* embryos at stage 10 demonstrating ectodermal marker (*ectodermin* and *foxi1a*) expression changes in Nup107*-* and Rest*-*depleted embryos. Three biological replicates were used for all experiments. All data represent results from experiments replicated at least three times in the laboratory. Statistical significance was determined using two-way ANOVA analysis. ****P<0.0001. ns, not significant. Scale bars: 500 μm.

*Rest* has two seeds that perfectly match the *miR427* consensus within its 3′ UTR, including an extended 8 nt reverse complement pairing AGCACTTT. Therefore, it has the potential to be directly targeted by *miR427*. We overexpressed mature *miR427* in wild-type embryos and detected a reduction in *rest* expression by whole-mount *in situ* hybridization (Fig. 6A). Additionally, we observed an upregulation of *rest* expression in embryos injected with *nup107* MO, which was consistent with the results from the RNA-seq experiment. Moreover, if we then expressed *miR427* in Nup107-depleted embryos, we could rescue *rest* mRNA expression. These findings supported the idea that *rest* could be a direct target of *miR427*.

Next, we examined whether *rest* played a role in early embryonic patterning of the ectodermal tissue. We performed whole-mount *in situ* hybridization in stage 10 embryos to detect the expression of two ectodermal markers, *ectodermin* and *foxi1a.* Consistent with our previous results, the depletion of Nup107 resulted in the upregulation of the expression of both ectodermal markers (Fig. 6B). However, depletion of *rest* in Nup107-depleted embryos rescued this phenotype. These results suggest that, besides neuronal patterning, *rest* could also contribute to ectodermal specification during germ layer differentiation.

Our results thus far suggest that depletion of Nup107 leads to a reduction in *miR427* levels, perdurance of *rest* as well as other maternal transcripts, and expansion of ectodermal fates and reduction of mesendodermal fates that dramatically alter gastrulation. We next returned to the question of why *miR427* levels were reduced in Nup107-depleted embryos. Following transcription from a locus spanning ∼100 kb in the *Xenopus tropicalis* v.9.1 genome, the primary miRNA transcript (pri-miRNA) undergoes cleavage in the nucleus into a 64 nt precursor miRNA (pre-miRNA) by the Drosha complex (Ha and Kim, 2014). The 64 nt precursor is then exported into the cytoplasm by exportin 5 (Yi et al., 2003) where it is cleaved by DICER into the mature 22 nt miRNA that can be loaded onto the RISC complex to execute target RNA degradation (Wang et al., 2011; Ha and Kim, 2014). As we detected lower levels of mature *miR427* in Nup107-depleted embryos (Fig. 4), we examined earlier steps in miRNA processing starting with the primary miRNA transcript (*pri-miR427*). Unexpectedly, in our RNA-seq ribozero timecourse, we saw no difference in the expression levels of *pri-miR427* when comparing Nup107-depleted embryos and controls (Fig. S9A,B). Therefore, the transcription of *pri-miR427* seemed to be intact. We next considered that aberrant processing of *miR427* could explain the reduction in the mature *miR427* in Nup107-depleted embryos. We assessed whether the mature *miR427* was captured in our ribozero depleted RNA-seq. We found that small fragments were very poorly captured by this protocol. There were 58,557 fragments of 22 bp out of 738 million total sequenced fragments (0.008%), of which we found two 22 bp fragments that could be assigned to *miR427*, thus we did not capture mature microRNA sequences in the ribozero sequencing (Fig. S9C).

**Fig. 7.**
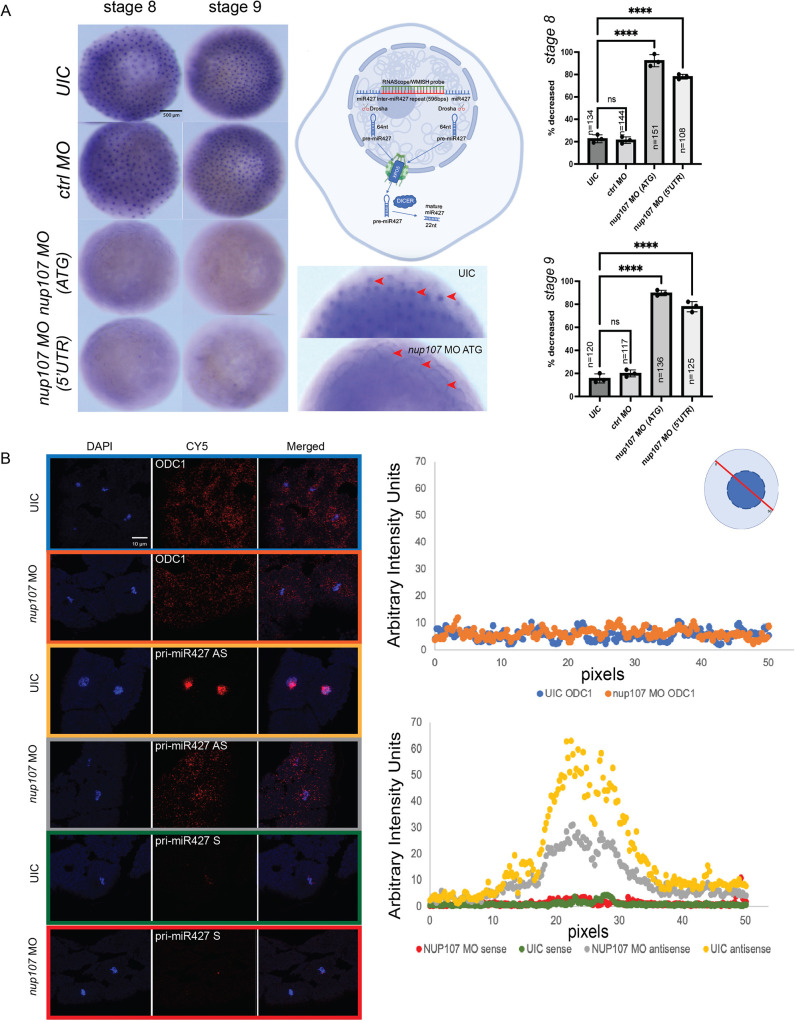
**The expression and subcellular localization of *pri-miR427*.** (A) Whole-mount *in situ* hybridization demonstrating the expression domain of *pri-miR427*. Higher magnification images at lower right reveal nuclear staining (red arrowheads). Cell diagram was created in BioRender by Kostiuk, V., 2025. https://BioRender.com/x94m509. This diagram was sublicensed under CC-BY 4.0 terms, and subsequently annotated. (B) (Left) Using RNAScope, subcellular localization of the *pri-miR427* (AS, antisense; S, sense) and *odc1* transcript (a housekeeping gene control). (Right) Signal quantification of *odc1* (top) and *pri-miR427* (bottom). The following number of embryos were used for this experiment: sense *pri-miR427* probe: 16 Nup107-depleted embryos and 20 control embryos; antisense *pri-miR427* probe: 20 Nup107-depleted embryos and 18 control embryos; antisense *odc1* probe: 19 Nup107-depleted embryos and 17 control embryos. The confidence intervals for measurements were: sense *pri-miR427* probe: *nup107* MO sample 1.3±0.6 (0.7-1.8), UIC sample 0.9±0.4 (0.6-1.3); antisense *pri-miR427* probe: *nup107* MO sample 10.3±3.4 (6.9-13.8), UIC sample 18.8±7.4 (11.4-26.2); antisense *odc1* probe: UIC sample 5.7±0.8 (4.9-6.5), *nup107* MO sample 6.3±0.8 (5.5-7.0). Three biological replicates were used for all experiments. All data represent results from experiments replicated at least three times in the laboratory. Statistical significance was determined using two-way ANOVA analysis. *P*<0.05 was considered statistically significant. ****P<0.0001. ns, not significant. Scale bars: 500 μm (A); 10 μm (B).

As pri-miRNA cleavage steps take place both in the nucleus and the cytoplasm, we first checked the nuclear localization of *pri-miR427*. *Pri-miR427* is one of the most abundantly transcribed RNAs in the early embryo, and we detected nuclear localization of the transcript by whole-mount *in situ* hybridization (Fig. 7A) (Owens et al., 2016). Importantly, this probe was designed against the 596 bp sequence found in the long primary transcript and does not recognize the shorter 64 bp pre-miRNA or 22 bp miRNA products (Fig. 7A). Examining the expression of the *pri-miR427*, we readily detected a punctate pattern in control embryos evocative of nuclear localization of the transcript (Fig. 7A). However, in Nup107-depleted embryos, the signal was more diffuse and did not accumulate in any obvious structure. To better resolve the localization of *pri-miR427*, we performed RNAScope (Wang et al., 2012a) *in situ* hybridization (Fig. 7B). In control embryos at Stage 8, *pri-miR427* transcripts were primarily detected in the nucleus, which was co-stained with DAPI. Strikingly, in Nup107-depleted embryos, the *pri-miR427* signal failed to accumulate in the nucleus and was instead distributed throughout the cell. We confirmed the specificity of this signal with a sense control probe. Importantly, the redistribution of *pri-miR427* was specific for this transcript, as *odc1* localization was not impacted by Nup107 depletion. Additionally, to test whether this was specific to *miR427*, we examined the subcellular localization of other transcripts in Nup107-depleted embryos. Following nuclear-cytoplasmic fractionation and RNA extraction, we quantified the levels of *odc1* and *u6*, known cytoplasmic and nuclear transcripts, respectively (Lange and Gerbi, 2000; Dhorne-Pollet et al., 2013). In both control and Nup107*-*depleted embryos, there were comparable levels of *odc1* and *u6* in the cytoplasmic and nuclear fractions only, respectively (Fig. S9D). In either Nup107-depleted embryos or controls, *odc1* was not detected in the nuclear fraction, nor did we find *u6* expression in the cytoplasm. This result supported the notion that Nup107 depletion has a specific effect on *pri-miR427* localization. Therefore, the sum of our data suggests that Nup107 is required to specifically retain *pri-miR427* in the nucleus.

## DISCUSSION

Our results expand an understanding of how differential and stage-specific expression of certain nups contributes to vertebrate development. Specifically, we detected a high level of Nup107 expression in pre-gastrula embryos in contrast to the ubiquitous expression of Nup62. Nup107 expression was also different than Nup188, which appeared in waves during the stages associated with ciliogenesis. A major challenge for the field is to decipher the functional significance of these unique patterns of nup expression in embryos and adult tissues and to translate this information to potential changes to NPCs themselves.

A key step in embryonic development is the maternal-zygotic transition, which requires the clearance of maternal transcripts and the activation of the zygotic genome. Based on the RNA-seq data, Nup107 is essential for the first step of this process: maternal transcript clearance. Specifically, our data support a model where Nup107 plays an important role in retaining *pri-miR427* in the nucleus, where it undergoes processing. *Pri-miR427* is zygotically expressed at high levels to help clear many maternal RNAs. To produce mature *miR427*, the *pri-miR427* must be processed in the nucleus by Drosha before nuclear export, as cytoplasmic Dicer is unable to process the *pri-miR427* (Ha and Kim, 2014). Therefore, the premature export of the *pri-miR427* leads to a loss of mature *miR427*, which fails to clear maternal mRNAs including *rest*, and leads to the upregulation of ectodermal cell fates at the expense of mesendodermal fates. We speculate that the loss of Nup107 from the NPC may impact the integrity of the NPC diffusion barrier established by FG-containing nups and might allow *pri-miR427* to diffuse out from the nucleus before processing.

A recent zebrafish study implicated another Y-complex protein and Nup107 binding partner, Nup133, in the maternal-to-zygotic transition (Shen et al., 2022). In contrast to Nup107 depletion in our study, loss of Nup133 affected the second step, zygotic genome activation. Therefore, it is possible that these studies describe complementary findings in which NPC composition could play a role first in maternal transcript clearance and later in zygotic genome activation. Regardless, the disruption of Y-complex components has a major impact on these key developmental events.

Our data suggest that abnormal nup expression leads to failure of proper *miR427* processing, with subsequent defects in germ layer differentiation. In fact, the Nup107 depletion phenotype, including germ layer specification and dorsal Organizer patterning, is identical to the targeted depletion of *miR427* described in *Xenopus laevis* and human embryonic stem cells (ESCs) (Rosa et al., 2009). Similar to our results, miR302, a human ortholog of frog *miR427*, promotes the mesendodermal lineage specification at the expense of neuroectoderm formation. Additionally, the depletion of *miR427* in *X. laevis* led to the disruption of Organizer formation and severe defects in dorsal mesodermal patterning. While these phenotypes in human ESCs and frog embryos align with our findings, we introduce a role for Nup107 in ensuring the proper localization of *pri-miR427*, which is crucial for its proper processing and function. A different study showed that the genetic deletion of the miR430 cluster in zebrafish resulted in phenotypes that are similar to our results in Nup107-depletion experiments (Liu et al., 2020). Specifically, the deletion of the miR430 cluster resulted in reduced expression of endodermal and mesodermal markers, including the dorsal mesoderm, and increased the expression of ventral mesodermal markers. Therefore, the similar phenotype amongst these studies supports the notion that Nup107 regulates the function of *miR427* in germ layer patterning.

Our study, as well as the recent zebrafish study focusing on Nup133, poses an important question – could the oocyte or early cleavage embryo have a structurally different NPC? The zebrafish study suggests that immature NPCs prevent the premature nuclear entry of maternal transcription factors and act as a timer for zygotic genome activation (Shen et al., 2022). Our study suggests that Nup107 is expressed early in development in order to retain *pri-miR427* in the nucleus, perhaps through a similar mechanism. Although the molecular composition and structure of the NPC is understood in the *Xenopus* oocyte (Zhu et al., 2022; Fontana et al., 2022; Zhang et al., 2020; Sellés et al., 2017), the implication is that either there are more copies of Nup107 in immature NPCs, or, that mature NPCs contain fewer copies of Nup107. It is also possible that the higher levels of Nup107 reflect a yet-to-be-defined role for Nup107 in a pool outside of NPCs, suggesting potential topics for future work.

Finally, the inspiration for our work came from the notion that mutations in NUP107, a crucial cellular protein, were identified in individuals with tissue-specific malformations. Through the process of patient-driven gene discovery, we identified a unique expression pattern of Nup107 in a vertebrate embryo with preferential mRNA and protein expression before the onset of gastrulation. We also demonstrated that the depletion of Nup107 disrupts the localization of *miR427*, leading to abnormal maternal transcript clearance. Moreover, Nup107-depleted embryos develop multiple downstream defects including abnormal germ layer patterning. Therefore, the distinct expression of Nup107 is crucial for its function in early development and encourages continued investigation of nups in development as a fruitful endeavor.

## MATERIALS AND METHODS

### *Xenopus* husbandry

*X. tropicalis* were raised, housed, and cared for according to established protocols that were approved by Yale Institutional Animal Care and Use Committee (IACUC). All animals used in this study were from the N (Nigerian) strain. To obtain embryos, we induced ovulation, performed *in vitro* fertilization, and raised embryos in 1/9× MR as previously described (del Viso and Khokha, 2012). Stages were determined using the Nieuwkoop and Faber standard table (Faber and Nieuwkoop, 1994).

### *Xenopus* embryonic injections and manipulations

Following standard protocols, we performed microinjections using *X. tropicalis* one-cell-stage fertilized embryos (Khokha et al., 2002). To deplete Nup107, CRISPR sgRNAs were designed using CRISPRSCAN with the v9.0 gene models of the *X. tropicalis* genome (Moreno-Mateos et al., 2015). The following sgRNA seeds were used to target the *nup107* locus: CR1 targeting *nup107* exon 3 (5′-GGTACAGACTCCTGGGCGTT-3′), CR2 targeting exon 12 (5′-GGCCTGGAGAGCAGCTACAT-3′), and CR3 targeting exon 23 (5′-GGACAGTGCTCTGCCAGCTG-3′). Then 2 nl of an injection mix consisting of 400 pg of sgRNA, 1.2 ng of Cas9 protein and Alexa-488 fluorescent tracer was incubated at 37°C for 10-15 min and injected into the one-cell embryo using a glass microneedle, as previously described (Khokha et al., 2002). Translation-blocking MOs were obtained from GeneTools LLC to deplete the following genes: *nup107* ATG blocking (10 ng/embryo, 5′-TACCGGCGAAAGCATATCCATGCTG-3′), *nup107* 5′ UTR blocking (10 ng/embryo, 5′-ATCAGAGAACTCGATTGGCTGGAT-3′), *rest* ATG blocking (10 ng/embryo, 5′-GGCCATGTTTATAACTTTTCAGGGA-3′), *nup133* ATG blocking (5 ng/embryo, 5′-CCGCTGTGTCGGGATAGTTTGAAAA-3′), *nup160* ATG blocking (10 ng/embryo, 5′-GCCATGTTTCATACTGGCAA-3′). A control morpholino targeting a human β-globin intron mutation that causes beta-thalassemia was obtained from GeneTools (5′-CCTCTTACCTCAGTTACAATTTATA-3′) and injected at 10 ng/embryo. A Human *NUP107* cDNA was obtained from DNASU Plasmid Repository (clone HsCD00860375 in pDONR221) and subcloned into the pCS2+ expression vector. To generate a full-length capped mRNA, plasmids were linearized using NotI and *in vitro* transcribed using the mMessage mMachine Sp6 Transcription Kit (Ambion) according to the manufacturer's protocol. Overexpression and rescue experiments were performed by injecting human *NUP107* mRNA (200-800 pg) into one-cell embryos with an mCherry mRNA fluorescent tracer. Protein expression of human *NUP107* mRNA was verified using western blot. We overexpressed miR427 by injecting one-cell *X. tropicalis* embryos with miRNA duplexes (5′-aaagugcuuucuguuuugggcg-3′) (100 fmol and 50 fmol) obtained from Integrated DNA Technologies.

### Genotyping

To confirm CRISPR-Cas9 cutting efficiency, we genotyped embryos using PCR amplification of the target site followed by ICE analysis (Conant et al., 2022). Genomic DNA was extracted from at least ten CRISPR-Cas9-injected embryos 72 hpf. The DNA locus containing the Cas9 cut site was amplified with Phusion High-Fidelity DNA Polymerase (New England Biolabs) using the following conditions: 98°C for 2 min, followed by 36 cycles at 98°C for 10 s, 62°C for 30 s and 72°C for 60 s. Final extension was at 72°C for 10 min. The length of PCR products was confirmed using gel electrophoresis. PCR products were purified with the PCR & DNA Cleanup Kit (Monarch) and sequenced with one of the PCR amplification primers. Insertion-deletion (indel) percentages and knockout scores were determined for all three sgRNAs using ICE analysis. The following primers were used for genotyping:

CR1-F 5′-GCTCGTGTTACAGAAGCACAAGCAG-3′; CR1-R 5′-CGCATCGGCTTGTTCATCCAATGG-3′; CR2-F 5′-GACTTAAGGTATGGAGACCCCATACCC-3′; CR2-R 5′-GGAGGATGATCAGACAGTTCGCC-3′; CR3-F 5′-GATCCTGCTCAGAGGGCGGAAG-3′; CR3-R 5′-GACATGGGAAGGCAGAACTGGGC-3′.

### Whole-mount *in situ* hybridization

Whole-mount *in situ* hybridization was carried out as previously described (Khokha et al., 2002). Briefly, embryos were collected at appropriate stages and fixed in 1× MEMFA with 4% formaldehyde overnight at 4°C, washed in PBS with 0.1% Tween 20 (PBST) and gradually dehydrated to 100% ethanol. Following rehydration, embryos were hybridized for at least 16 h at 65°C with digoxigenin-labeled antisense RNA probes. Sense and antisense probes were generated using Sp6 and T7, respectively, with the HiScribe High Yield RNA Synthesis Kit (E2070S and E2050S) from New England Biolabs according to the manufacturer's instructions. Plasmids used for probe synthesis are shown in Table S1.

After probe hybridization, embryos were washed, blocked and then incubated with anti-DIG-Fab fragments (Roche) for 4 h at room temperature. mRNA expression was detected using BM purple (Sigma-Aldrich). To stop the colorimetric reaction, embryos were fixed in 4% paraformaldehyde with 0.1% glutaraldehyde in 1× SSC. Embryos were then bleached until pigment was eliminated. Images were taken using a Canon EOS camera (DS126201) mounted on a Zeiss Discovery V8 stereomicroscope. To score embryos, we defined the signal detected in the majority of the control embryos as the normal signal and scored all embryos compared to this baseline. A representative image of the control embryo is shown in each figure.

### Western blot

Proteins were extracted from whole-cell extracts derived from embryos using RIPA buffer supplemented with Halt protease and phosphatase inhibitors (Thermo Fisher Scientific, 78440). Protein concentrations were determined using a Bradford reagent (Bio-Rad, 5000201) and bovine serum albumin standards. Then, 4-12% NuPAGE Bis-Tris Bolt gels were used to separate proteins, which were transferred to nitrocellulose and blocked in 5% milk solution in Tris-buffered saline with 0.1% Tween 20 (TBST). The primary antibodies (Table S2) were used followed by HRP-conjugated secondary antibodies. Antibody labeling was visualized by HRP-generated ECL using Thermo Fisher Scientific Pierce ECL Western Blotting Substrate and an Azure 200 gel imager (Azure Biosystems).

### Northern blot

Total RNA was extracted from embryos using Trizol (Thermo Fisher Scientific, 15596026). We loaded 10 µg of RNA on to a 6% polyacrylamide gel. Total RNA loading levels were detected by staining the polyacrylamide gel with 0.5 µg/ml of ethidium bromide as previously described (Nicoli et al., 2012). By blotting overnight at 4°C, the RNA was transferred to the AM10102 BrightStar™-Plus Positively Charged Nylon Membrane, which was then crosslinked using a Stratalinker (SS-UV1800). Pre-hybridization was carried out using ULTRAhyb Oligo Buffer (Ambion, AM8663) at 42°C for 30 min. Biotin-labeled DNA probes complementary to the RNA sequences of interest were diluted 1:1000 in the ULTRAhyb Oligo Buffer to perform the hybridization step overnight at 42°C. The following oligo was used to probe for miR427: 5′-ACGCCCAAAACAGGAAGCACTTT-3′ (Yamaguchi et al., 2014). Washes were carried out at 42°C using a wash buffer containing 2× SSC and 0.01% SDS. Signal development and detection were performed using the Chemiluminescent Nucleic Acid Detection Module Kit according to the manufacturer's instructions (Thermo Fisher Scientific, 89880). Images were obtained using the Azure 200 gel imager (Azure Biosystems).

### Nuclear and cytoplasmic RNA extraction

Subcellular fractionation to isolate nuclear and cytoplasmic fractions was performed following a previously published protocol (Sondalle et al., 2019; Chamousset et al., 2010). Briefly, embryos were washed and resuspended in ice-cold 1× PBS before centrifuging at 4°C at 220 ***g*** for 5 min. After removing supernatant, cells were placed on ice and resuspended in ice-cold 1× PBS. The cellular suspension was spun at 220 ***g*** at 4°C for 5 min. Cells were then resuspended in 5 ml of Nuclei Harvesting Buffer containing N-ethylmaleimide and EDTA-free protease inhibitors and incubated on ice in a cold room for 10 min. Swollen cell suspension was then homogenized using a douncer on ice until most of the cells were burst with intact nuclei. Dounced cells were then centrifuged at 220 ***g*** at 4°C for 5 min. The supernatant containing the cytoplasmic fraction was then removed and processed for RNA extraction using Trizol LS reagent (Thermo Fisher Scientific, 10296010) based on the manufacturer's instructions. The pellet at the bottom containing the nuclei was processed using Trizol reagent (Thermo Fisher Scientific, 15596026) following the manufacturer's protocol.

### qRT-PCR

#### MicroRNA

Total RNA, including miRNA, was isolated from stage 8 embryos using QIAzol Lysis Reagent (QIAGEN, 79306) followed by purification according to the instructions from the miRNeasy kit (QIAGEN, 217084). We used 1 µg of RNA for cDNA synthesis, which was performed using miRCURY LNA RT Kit (QIAGEN, 339340). miRCURY LNA SYBR Green PCR Kit (QIAGEN, 339345) was used to perform the qRT-PCR experiment. LNA probes for *miR427* and *U6* were synthesized according to the miRCURY LNA miRNA Custom Probe Assay and were ordered from QIAGEN. Overexpression of *miR427* served as a positive control. Quantifications were performed using the delta-delta Ct method (Livak and Schmittgen, 2001).

#### Nuclear and cytoplasmic RNA transcripts

Nuclear and cytoplasmic RNA was purified from stage 8 embryos using Trizol and Trizol LS reagents, respectively (Thermo Fisher Scientific, 15596026 and 10296010). We used 1 µg of RNA for cDNA synthesis using iScript cDNA Synthesis Kit (Bio-Rad, 1708890). QuantiTect SYBR Green PCR Kit (QIAGEN, 204143) was used to perform the qRT-PCR experiment. The following primers were used for amplification: *odc1* (forward 5′-GGAAAGCAACCTTGGCAAGA-3′, reverse 5′-AGGCCACACTGGCAACTCA-3′) (Dhorne-Pollet et al., 2013) and *u6* (forward 5′-GTGCTTGCTTCGGCAGC-3′, reverse 5′-GCTTCACGAATTTGCGTGTC-3′). Quantifications were performed using the delta-delta Ct method (Livak and Schmittgen, 2001).

### RNAScope assay

Stage 8 embryos were collected and fixed in 4% paraformaldehyde in PBS at 4°C overnight. Embryos were washed in 70% RNAse-free ethanol, placed into Epredia HistoGel Specimen Processing Gel (Thermo Fisher Scientific, Epredia HG4000012), and embedded into paraffin to generate 5 μm slices. RNAScope Multiplex Fluorescent Assay (Advanced Cell Diagnostics, 323136) was performed according to the manufacturer's instructions. Three probes were generated by Advanced Cell Diagnostics, a Bio-Techne company: *pri-miR427* antisense (1212691-C1), *pri-miR427* sense (1223871-C1), and ODC1 (1214511-C1). Additionally, both sense and antisense *pri-miR427* probes were designed to target the 596 bp region within the primary miRNA transcript (Chr. 03: 4010201-4010797) that is not present in the pre-miRNA or the mature miRNA. DAPI staining was used to visualize the nucleus. ProLong Gold Antifade reagent was applied to samples (Thermo Fisher Scientific, P10144). Samples were imaged using an LSM 880 Airyscan confocal microscope (Zeiss). Images were analyzed using Fiji software to detect the signal along a straight line traversing the cytoplasm and the nucleus.

### RNA-seq

Synchronized embryos were generated by *in vitro* fertilization as described above. UIC and embryos injected with 10 ng of the ATG *nup107* MO were raised in 1/9× Modified Ringer's solution at 25°C and then ten embryos were collected from each group at 30 min intervals from stage 6 until stage 12. We added 10 µl of an ERCC RNA Spike-In Mix (Thermo Fisher Scientific) to 860 µl of DEPC-treated water and then diluted 1:10 into Trizol. Ten embryos per timepoint and condition (UIC and MO) were homogenized in 100 µl of Trizol+Spike-In mixture. Each sample was frozen using ethanol and dry ice and then stored at −80°C. All samples were processed for RNA extraction at the same time. Following isopropanol precipitation, RNA pellets were resuspended in DEPC-treated water and precipitated with 5 M LiCl at 4°C overnight. This solution was centrifuged at 4°C, and RNA pellets were washed twice with 70% ethanol. Final RNA products were resuspended in DEPC-treated water (2 µl/embryo). Ribosomal RNA depletion was performed using KAPA RNA HyperPrep Kit with RiboErase (HMR) (Roche, KR1351). Then, 150 bp paired end sequencing was performed on an Illumina NovaSeq6000.

### RNA-seq quantification

Stranded paired-end polyA+ and ribozero 150 bp RNA-seq reads were aligned to the Xt9.1 genome combined with ERCC spikes using STAR (Dobin et al., 2013) and quantified as transcripts per million (TPM) for each isoform with RSEM using the RSEM-STAR pipeline, with additional options ‘--seed 1618 --calc-pme --calc-ci --estimate-rspd --paired-end’ (Li and Dewey, 2011). This quantified 34,284 genes, which we filtered to find those with sufficient temporal expression for further analysis. We selected genes that had runs of six consecutive samples with uncorrected TPM>0.4 in each of polyA+ and ribozero timecourses. This resulted 13,917 genes, with which we found excellent coherence between samples and no evidence of outlying samples by both Spearman Correlation and principal components analysis (Fig. S1A,B).

### RNA-seq differential expression analysis

To determine genes that are temporally differentially expressed, we used Gaussian process (GP) regression as we have previously applied (Owens et al., 2016; Sempou et al., 2022). All GP regression was performed with GaussianProcesses.jl (https://github.com/STOR-i/GaussianProcesses.jl; https://arxiv.org/abs/1812.09064). Due to the overdispersed nature of RNA-seq count data, we apply a variance stabilizing transform that puts all genes on the same scale: 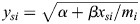, with *x*_*si*_ the dinucleotide corrected abundance of gene *i* in sample *s*, *m*_*i*_ the maximum *x*_*si*_ over all samples, and *α*=1,  *β*=1000. We then perform exact GP regression (GP prior and a Gaussian likelihood) with Matern52 kernel, we optimize the three associated hyperparameters: 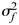 the signal variance, *τ* the timescale (using previous terminology, Owens et al., 2016; this parameter is commonly referred to as the lengthscale ℓ), and 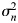, the sample noise variance. Parameters are selected by optimizing marginal log-likelihood with parameters in log space: log*σ*_*f*_, log*τ*, log*σ*_*n*_, and to ensure physiologically reasonable values for each, we place GP priors, 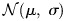 over each of these variables respectively 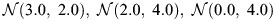. We optimize the log marginal likelihood by LBFGS followed by an additional Nelder Mead optimization step to help avoid local optima. Finally, we report GP median and 95% confidence intervals through our inverted data transformation:

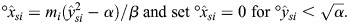
To determine temporal differential expression, we calculate a marginal likelihood ratio for whether we prefer separate GP models for UIC and *nup107* MO or a single GP model for all data combined. If *L*_*U*_ and *L*_*M*_ are the marginal log-likelihoods for UIC and *nup107* MO, respectively, and *L*_*UM*_ is the marginal log-likelihood for a single regression through UIC and *nup107* MO together, then we calculate log-likelihood ratio *LR*=*L*_*U*_+*L*_*M*_−*L*_*UM*_ of evidence in favor of two models (essentially that the UIC and *nup107* MO have different expression trajectories). This resulted in 959 and 705 genes differentially expressed for polyA+ and ribozero, respectively.

### Clustering

To determine sets of differentially expressed genes with similar trajectories, we applied K-means clustering to activated and repressed genes independently. We define a gene as increased if GP median for *nup107* MO exceeds UIC on average, and decreased if it does not. We cluster UIC and *nup107* MO genes by taking GP medians and normalizing by the maximum value experience by UIC or *nup107* MO. We then cluster both trajectories together, employing the k-means function offered by Clustering.jl (https://github.com/JuliaStats/Clustering.jl) with default settings and random seed 1618033. To determine the cluster number, we calculated the silhouette score for increased and decreased clusters for k=2-10. We found that k=3 was the mode of maximized silhouette scores for polyA+ and ribozero sequence for increased and decreased genes.

### 3′ UTR motif and microRNA seed enrichment

We assessed the enrichment of 3′ UTR motifs EDEN (UAUAUAUGUGUGUCUAUCGUCACUUGUAUGUCAAAUAUU; Paillard et al., 1998); ARE (AUUA; Voeltz and Steitz, 1998) and YTHDF2 (RRACH; Zhao et al., 2017) within clusters of differentially expressed genes. We counted compatible instances within each 3′ UTR for each isoform of EDEN, ARE, and YTHDF2 motifs. We calculate enrichment by the association of at least one copy of a motif in a gene and a gene being present in a given cluster by Fisher’s exact test, reporting right-tail *P*-values. To calculate the enrichment of microRNA seeds, we counted the total number of perfect instances of all *X. tropicalis* microRNA seeds from miRbase (Kozomara et al., 2019) in the 3′ UTRs of each isoform of each gene, allowing us to count the total number of seed instances in 3′ UTR sequences for every gene. To calculate enrichments, we performed Fisher’s exact test to calculate the association between a gene having at least *N* copies of a microRNA seed and being present in a given cluster. We report all microRNA seeds as sequence present in transcriptome sequences, i.e. the reverse complement of the microRNA sequence exchanging U for T.

To identify mismatch or offset matches to the *miR427* sequence, we counted the minimum number of mismatches to an extended seed region corresponding to the first 11 nt *miR427* sequence: GAAAGUGCUUUcuguuuugggcg. In the reverse complement space we calculate the minimal number of mismatches to AAAGCACTTTC.

### Transcription factor motif enrichment in promoters

We identified genes associated with ‘regulation of transcription’ gene ontology terms in cluster U1. This provided a list of 52 genes. Of these, we were able to obtain cognate binding motifs for seven genes from the JASPAR database (Castro-Mondragon et al., 2022) and ISMARA (Balwierz et al., 2014): *arid4b* (motif Arid3b MA0151.1), *bbx* (UN0113.1), *hivep1* (ISMARA), *mga* (MA0801.1), *rest* (MA0138.1), *znf462* (UN0616.1), *znf518A* (UN0199.1). We identified instances of these motifs within the 500 bp upstream of promoters of genes in each cluster. We calculated the maximal motif score (a likelihood ratio between the motif and a background model of A, C, G, T frequencies in the Xt9 genome) over all promoters for a given gene. To identify the potential of these seven transcription factors to drive gene expression in any of the clusters, for each motif we selected the motif score threshold >5 that maximized the association between a gene having an above threshold motif and being present in any cluster by Fisher’s Exact test. For the seven genes, this resulted in motif score thresholds which we report with the maximal matching score (ms) for a motif: *arid3a*: 8.8 (ms: 9.09); *bbx*: 5.77 (ms: 20.16); *hivep1*: 17.42 (ms: 19.46); *mga*: 13.02 (ms: 14.13); *rest*: 10.31 (ms: 32.65); *znf462*: 16.35 (ms: 16.7); *znf518a*: 5.0 (ms: 15.84). We then calculated enrichments for all clusters at the selected score threshold.

### Quantification of *miR427* locus and *miR427* seed sequences in RNA-seq reads

We quantify the ribozero RNA-seq signal at the highly repetitive *miR427* locus as we have previously (Owens et al., 2016). We identify reads that ambiguously align to a ∼100 kb region within Chr. 03:133185000-133320000 in the *X. tropicalis* v9.1 genome assembly, we retain all reads that mapped ambiguously within this locus. We count the total of these reads to quantify *pri-miR427* signal and to generate a heatmap across the locus we increment read piles at all positions a given read aligns, such that for a read that aligns *N* times we increment the pile 1/*N* at all alignment positions within the *miR427* locus. To quantify potential *miR427* mature microRNAs within ribozero RNA-seq, we trimmed paired end reads by aligning read pairs to discover regions of reverse complementarity surrounded by Illumina sequencing adapters using https://github.com/owensnick/SequenceTrimmer.jl to identify fragments shorter than the 150 bp read length. We then counted instances of mature *miR427* sequence GAAAGTGCTTTCTGTTTTGGGCG – we found that short RNAs were very poorly captured: we identified two instances at 22 bp, two at 23 bp and four at 24 bp across 738 million sequenced fragments.

### Statistical analysis

Three biological replicates were used for all experiments, and statistical significance was determined using two-way ANOVA analysis. *P*<0.05 was considered statistically significant. All statistical analyses and graphs were generated using Prism software (version 9). Models were created using BioRender.com.

## Supplementary Material



10.1242/develop.202865_sup1 Supplementary information
