# Supplementary material for: Nup107 contributes to the maternal-to-zygotic transition by preventing the premature nuclear export of pri-miR427
Source: Development. 2025 Feb 4;152(2):dev202865. doi: 10.1242/dev.202865 (PMC11829755; doi:10.1242/dev.202865)
Supplement: Supplementary information [file develop-152-202865-s1.pdf]

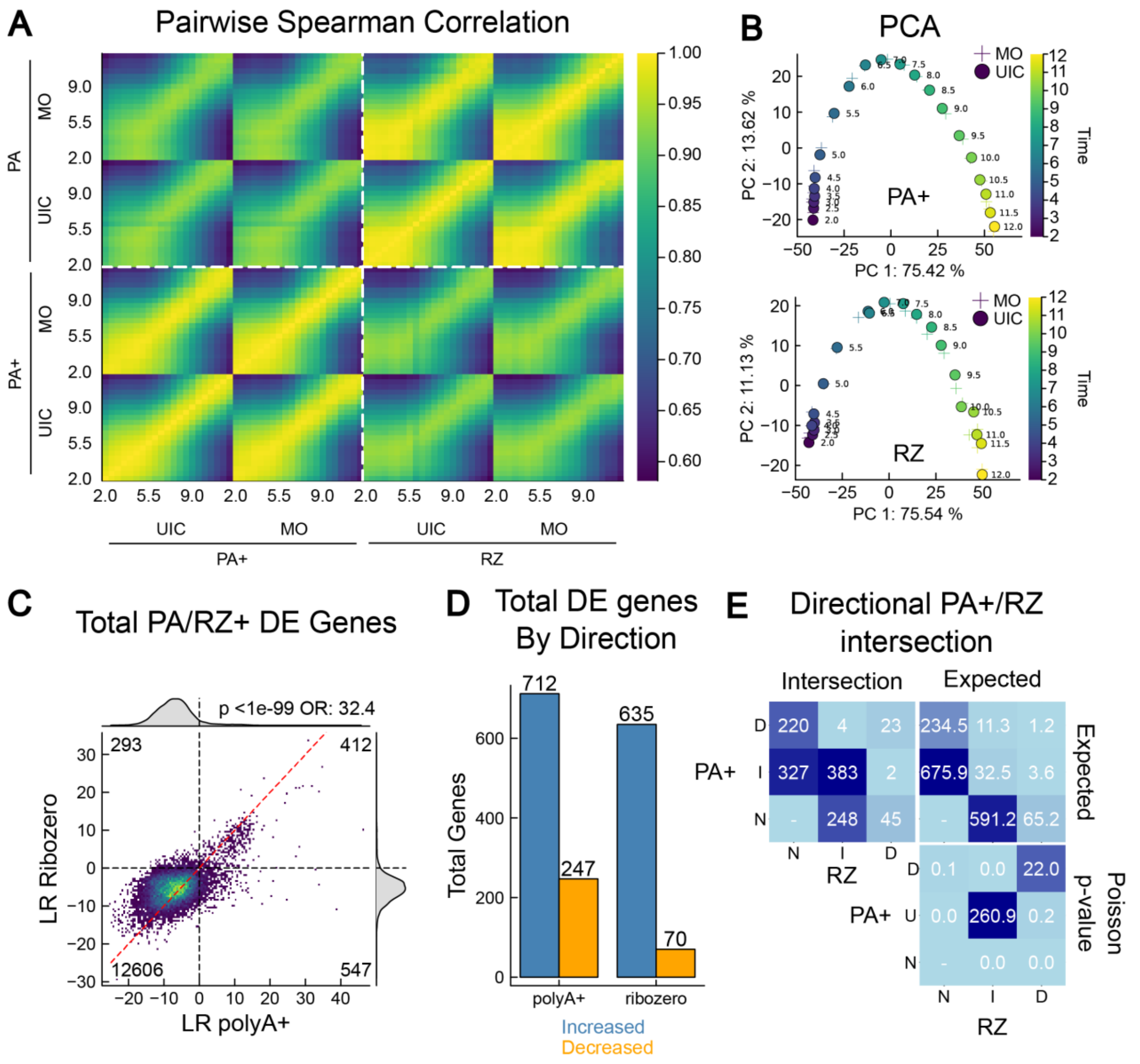

Fig. S1. Summary of quality and differential expression of polyA+ and ribozero RNA-seq timecourses.

A) Pairwise spearman correlation between all genes satisfying the non-zero run condition given in methods in both polyA+ (PA+) and ribozero (RZ) sequencing, color gives spearman correlation coefficient.

Development • Supplementary information

B) Principal components analysis (PCA) of all log TPM gene expression for all genes satisfying non-zero run condition, color gives time of sample, UIC = uninjected control, MO = *nup107* MO. Top gives PolyA+ and bottom gives ribozero.

C) Two-dimensional histogram, showing distribution of Gaussian process log-likelihood ratio (LR) scores for polyA+ and ribozero. LR > 0 defined as differentially expressed. Numbers give total genes in each quadrant, and p-value and odds ratio gives Fisher exact association for a gene being differentially expressed in both polyA+ and ribozero sequencing.

D) Total differentially expressed genes for polyA+ and ribozero by direction.

E) Directional agreement between polyA+ (PA+) and ribozero (RZ) sequencing, top left gives 3x3 contingency table for increased (I), decreased (D) and not differentially expressed (N), top right gives the expected intersection, and bottom right gives –log10 Poisson/Binomial p value right-tail for observed exceeding expected.

Development • Supplementary information

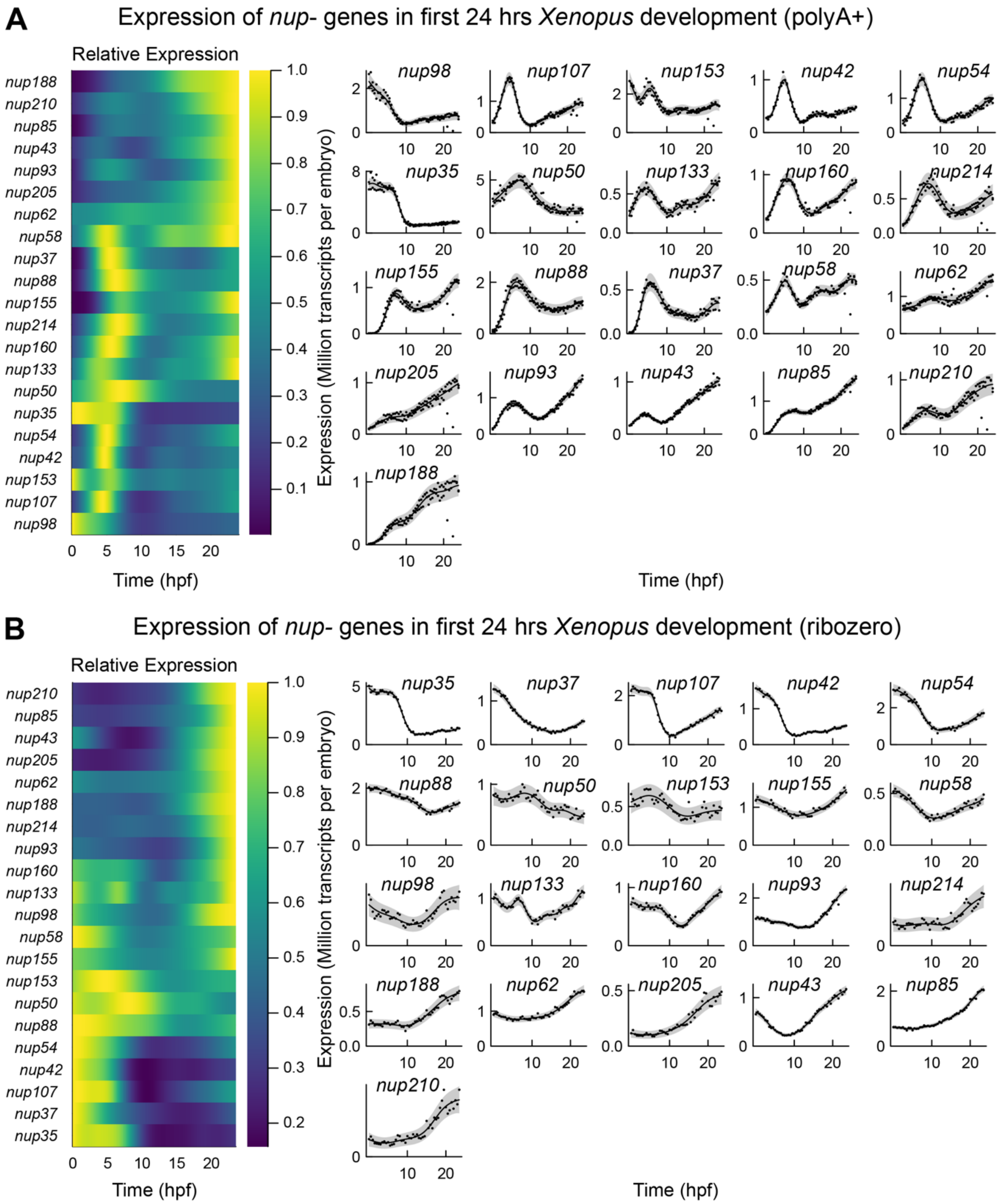

Development • Supplementary information

Fig. S2. Temporal expression of nup genes in *Xenopus* development.

Expression trajectories of all quantified *nup* genes detected in *Xenopus tropicalis* RNA-seq time series.[22] (Left) Heatmap of relative expression ordered by expression similarity, (right) expression trajectories in absolute transcripts per embryo with Gaussian process median and 95% confidence intervals as calculated in the original study.

A) polyA+ RNA-seq first 24 hours post fertilization (hpf).

B) ribosomal RNA depletion ribozero.

Development • Supplementary information

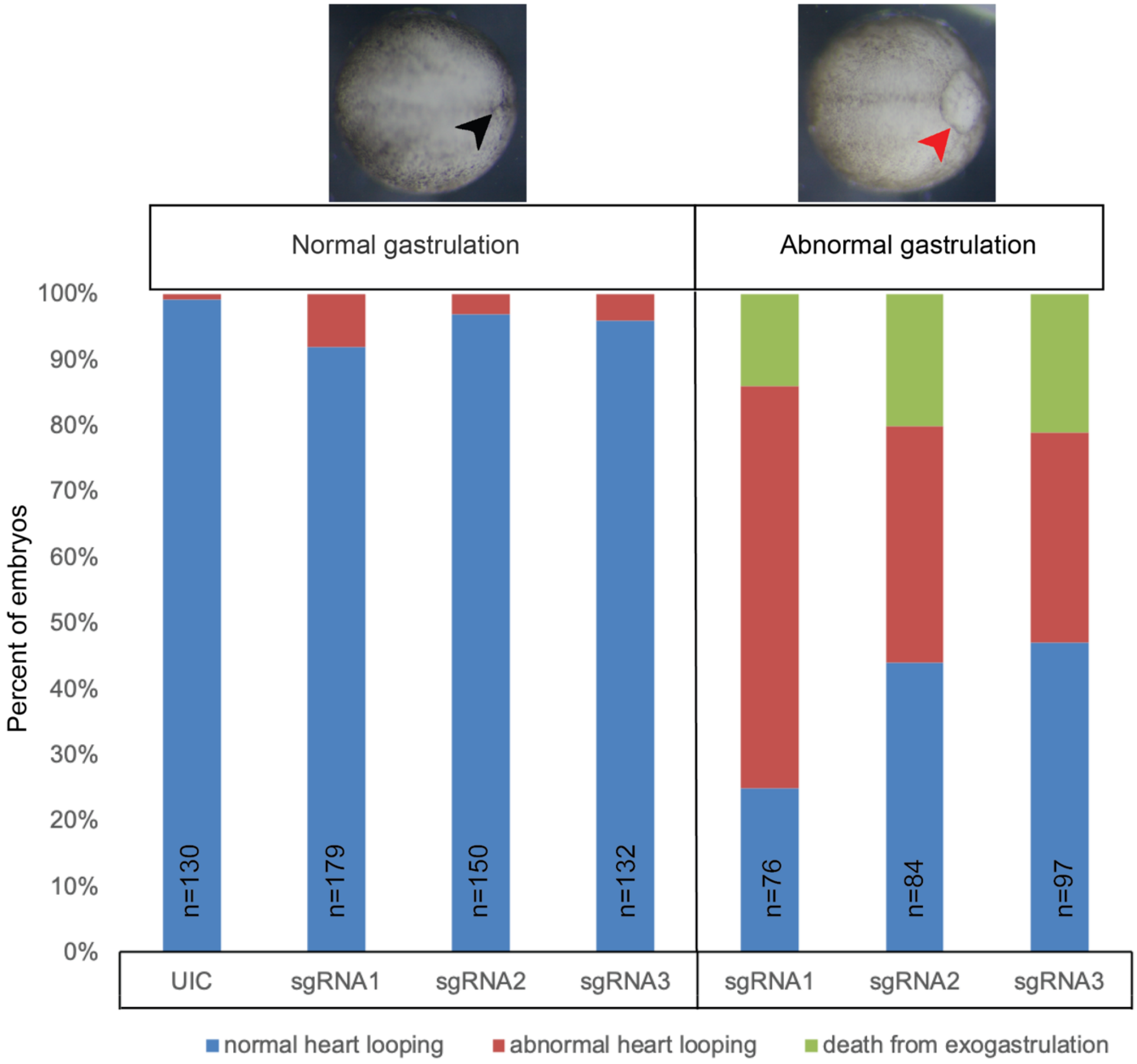

Fig. S3. The comparison of abnormal heart looping in embryos with normal (complete closure of the blastopore lip, black arrow) and abnormal gastrulation (incomplete closure of the blastopore lip, red arrow). Three biological replicates were used for all experiments. All data represent results from experiments replicated at least 3 times in the laboratory.

Development • Supplementary information

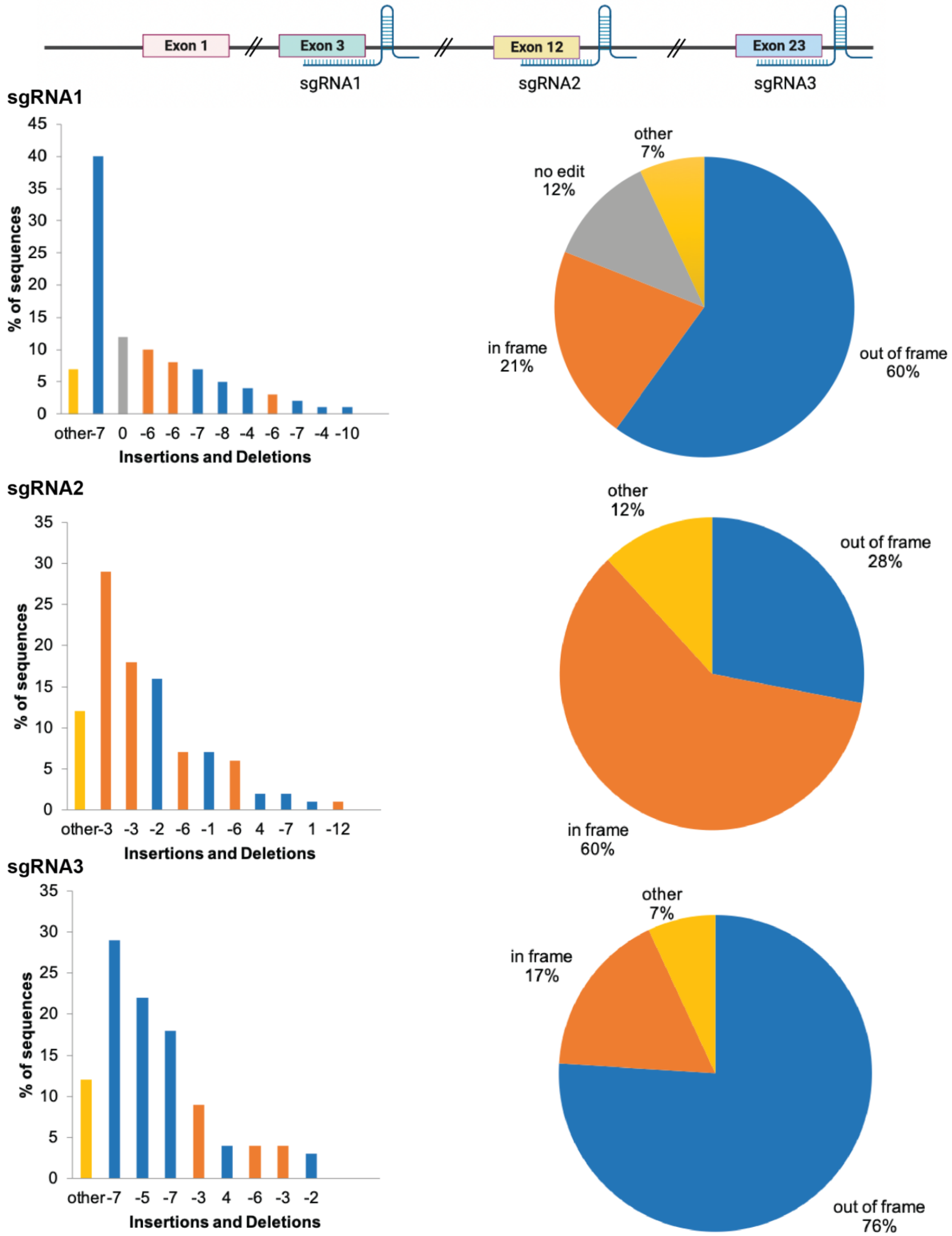

Development • Supplementary information

Fig. S4. ICE analysis of *nup107* crispants.

Schematic depicting sgRNAs targeting exons 3,12, and 23 (upper panel), and ICE analysis to determine *nup107* gene locus editing by CRISPR-Cas9. Three biological replicates were used for all experiments. All data represent results from experiments replicated at least 3 times in the laboratory.

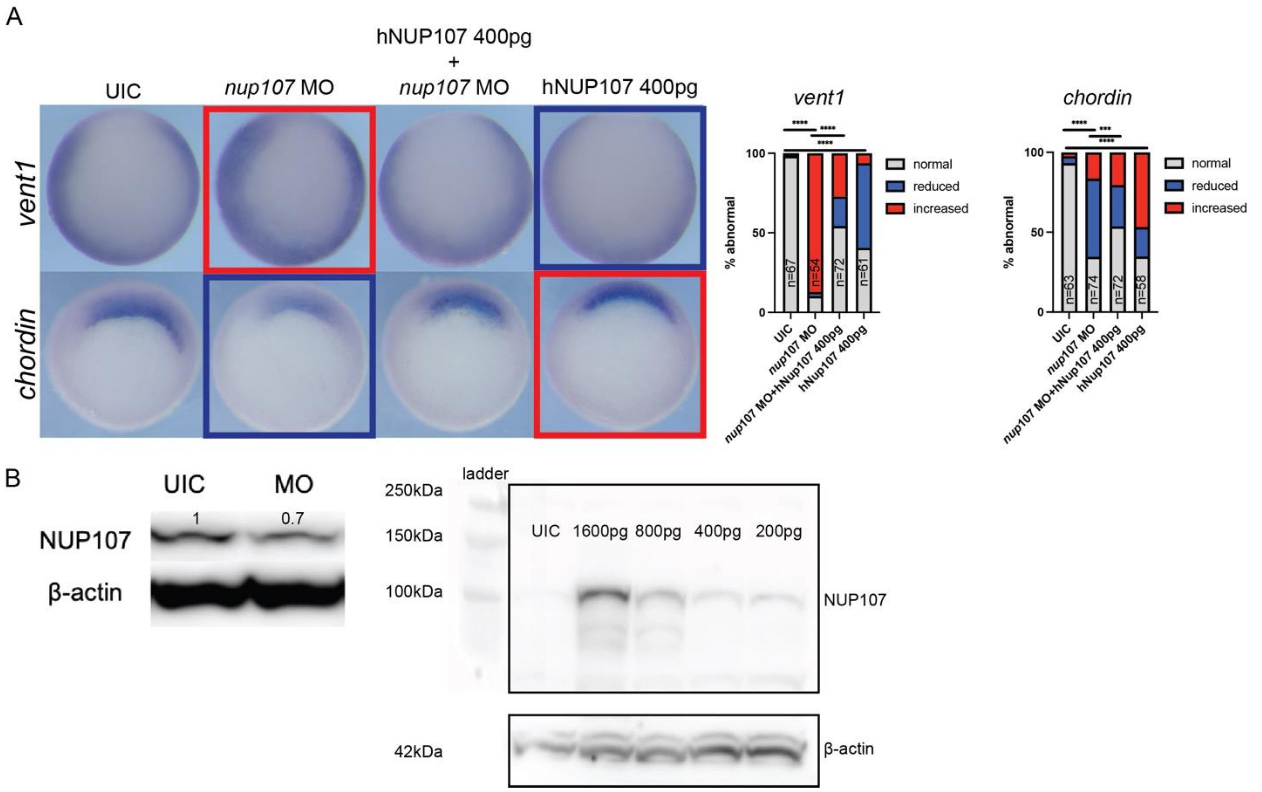

Fig. S5. Abnormal mesodermal patterning in Nup107-depleted embryos is rescued by human NUP107 mRNA expression.

A) Human NUP107 mRNA rescue of abnormal phenotypes associated with Nup107 depletion in ventral (*vent1*) and dorsal (*chordin*) patterning.

B) Western blots demonstrating the depletion of Nup107 protein (left) and the overexpression of human NUP107 mRNA (right) in *Xenopus tropicalis* embryos at stage 10.

Three biological replicates were used for all experiments. All data represent results from experiments replicated at least 3 times in the laboratory. Statistical significance was determined using Fisher Exact test analysis. $p<0.05$ was considered statistically significant. All statistical analyses and graphs were generated using Prism software (version 9).

Development • Supplementary information

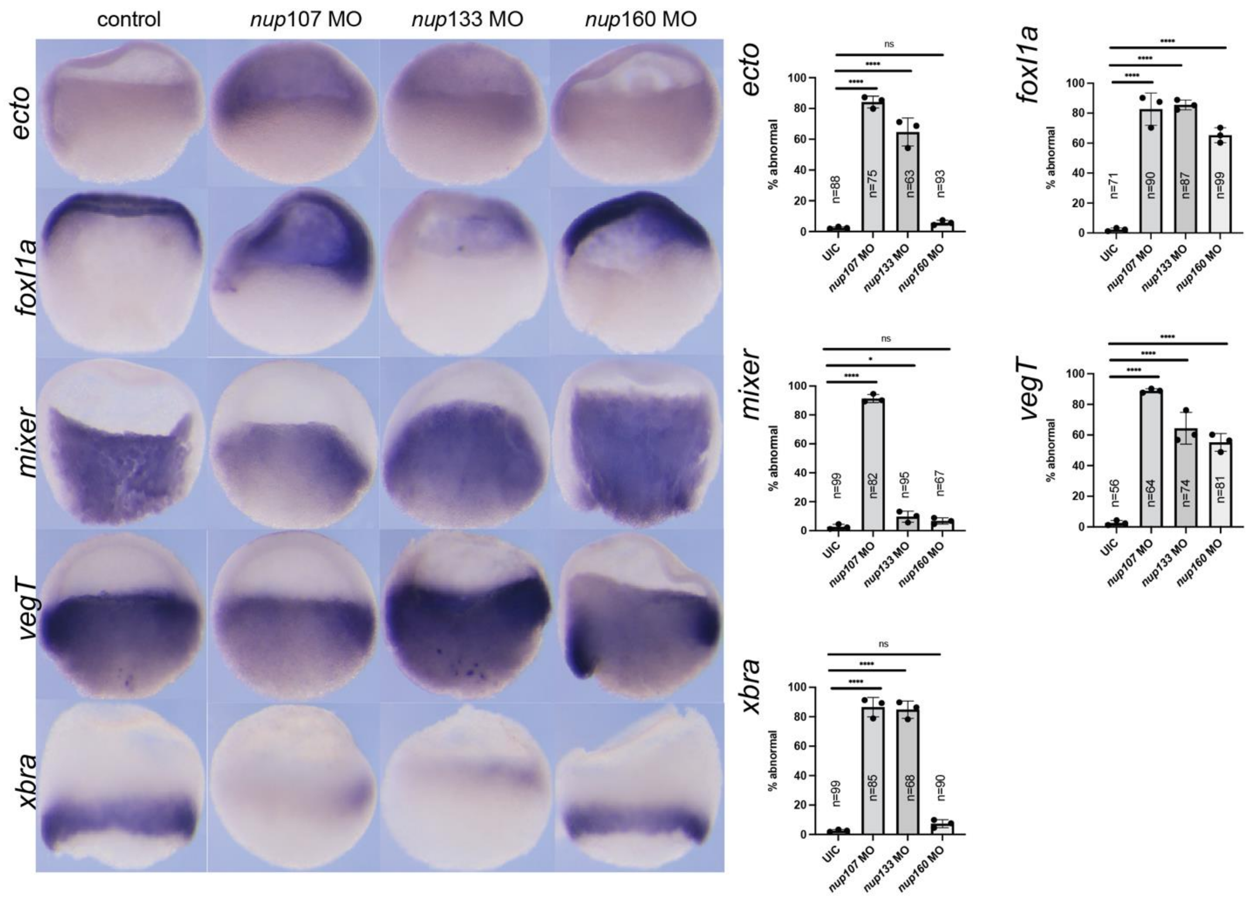

Fig. S6. The effects of outer ring nucleoporin depletion on germ layer patterning. Whole mount *in situ* hybridization showing germ layer expression patterns for the depletion of outer ring nucleoporins Nup107, Nup133, and Nup160. *Ecto* and *foxl1a* = ectodermal markers, *mixer* and *vegT* = endodermal markers, and *xbra* = mesodermal marker. Three biological replicates were used for all experiments. All data represent results from experiments replicated at least 3 times in the laboratory. Statistical significance was determined using two-way ANOVA analysis. $p<0.05$ was considered statistically significant. All statistical analyses and graphs were generated using Prism software (version 9).

Development • Supplementary information

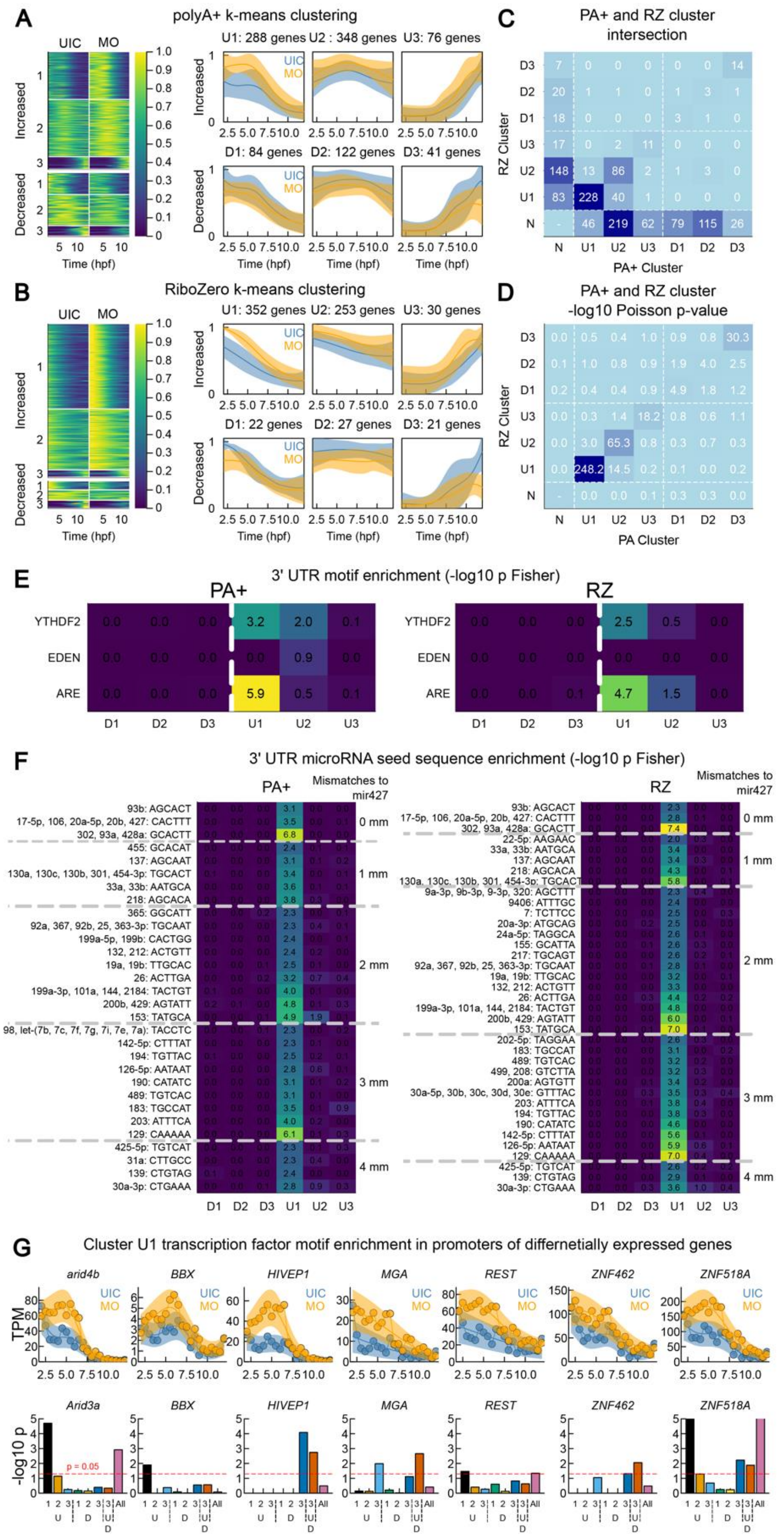

Development • Supplementary information

Fig. S7. Clusters of temporally differentially expressed genes in polyA+ and ribozero sequencing.

A, B) Summary of differentially expressed clusters for polyA+ (**A**) and ribozero (**B**) sequencing, divided into genes with increased or decreased expression on average. Visualization as in Figure 3AB. Left panel gives max normalized heatmap and right panel shows cluster mean ± SD. UIC = Uninjected Control, MO = *nup107* MO.

C) Intersection between polyA+ (PA+) and ribozero (RZ) clusters 7x7 contingency table, U1-3 = increased clusters 1-3, D1-3 is decreased clusters, N = not differentially expressed.

D) –log10 Poisson/Binomial right-tail p value for observed intersection exceeding expected, dominant intersection is in cluster U1.

E) Enrichment of given motif within the 3' UTRs of genes in each cluster for polyA+ (PA+) and ribozero (RZ). Color and numbers give –log10 Fisher exact right tail p value.

F) Enrichment of at least one copy of given microRNA seed within the 3' UTR of each cluster for polyA+ (PA+) and ribozero (RZ). Color and numbers give –log10 Fisher exact right tail p value. All seeds enriched $p < 0.01$ shown. Vertical labels give seed and microRNAs that share the same seed, e.g 427 = miR427. Seeds organized by number of mismatches to miR427 sequence.

G) Enrichment of polyA+ cluster U1 transcription factor motifs in the promoters of differentially expressed genes by cluster. Top gives gene expression in TPM for each gene for uninjected control (UIC) and *nup107* MO (MO), data points are sample TPM and line and shaded region are transformed Gaussian process median and 95% CI. Bottom gives –log10 Fisher exact right-tail p values for the association of at least one motif present in a promoter and the gene being present in the given cluster combination for polyA+ sequencing. 3UD = either cluster 3 in increased and decreased genes, the two zygotic response clusters, all = all differentially expressed genes.

Development • Supplementary information

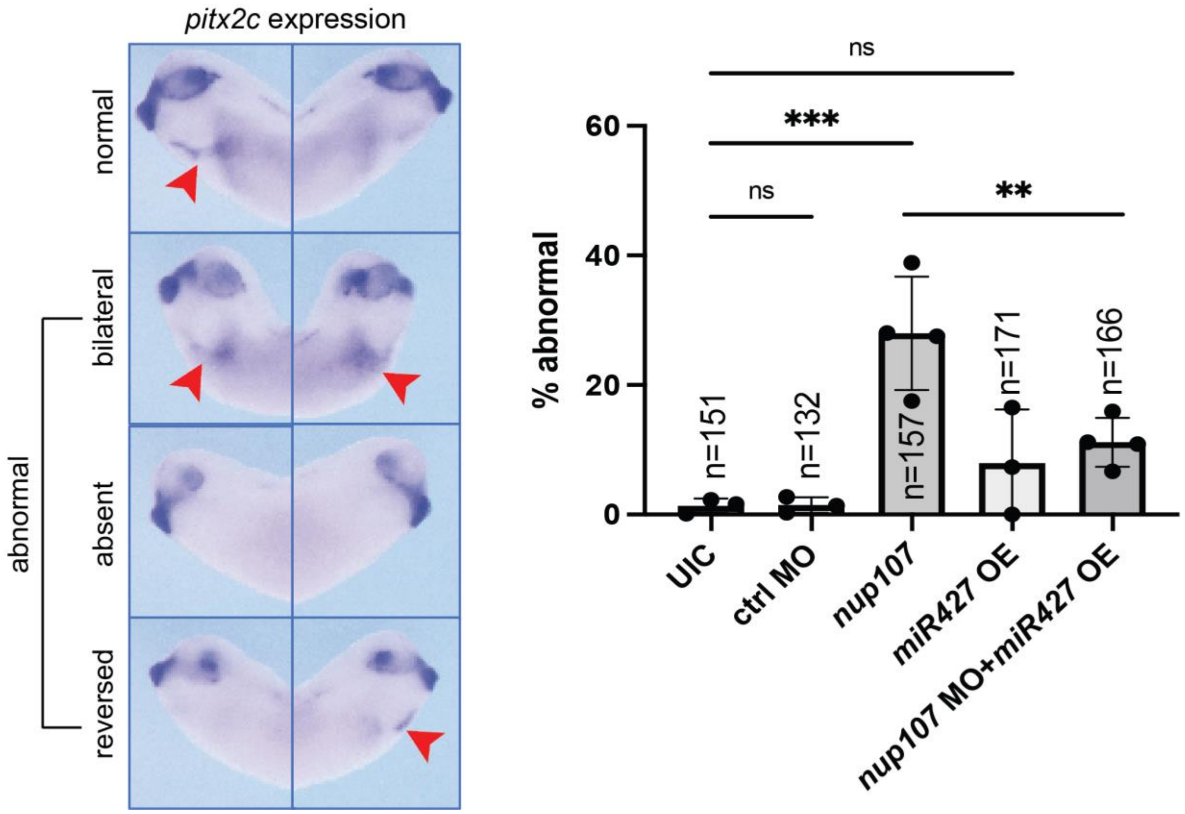

Fig. S8. Abnormal *pitx2c* expression in Nup107-depleted embryos is rescued by *miR427* overexpression.

Whole mount *in situ* hybridization showing *pitx2c* expression patterns. *Pitx2c* is normally expressed in the left lateral plate mesoderm, whereas abnormal expression manifests as right-sided, absent, or bilateral signal localization.

Three biological replicates were used for all experiments. All data represent results from experiments replicated at least 3 times in the laboratory. Statistical significance was determined using Fisher Exact test analysis. $p<0.05$ was considered statistically significant. All statistical analyses and graphs were generated using Prism software (version 9).

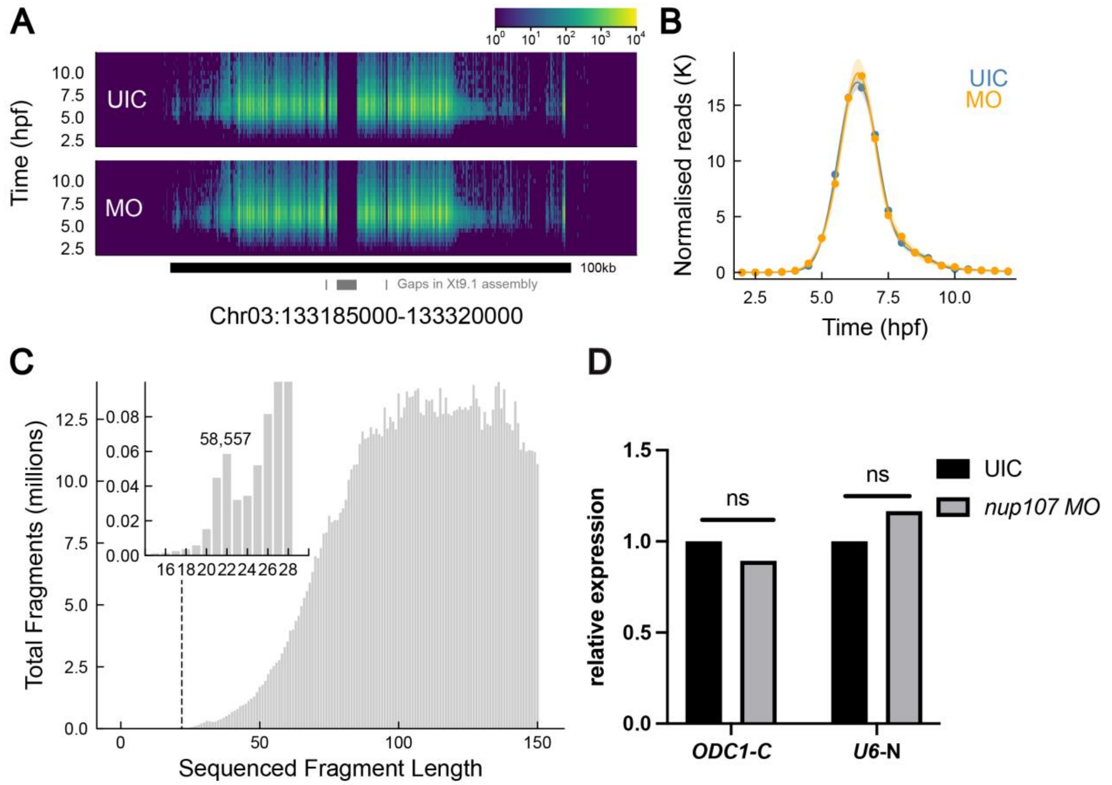

Fig. S9. Quantification of *pri-miR427* in ribozero timecourse.

A) heatmap of RNA-seq reads in uninjected control (UIC) and *nup107* MO timecourses. Reads correspond to *pri-miR427*, see **C)**, reads that map ambiguously within *miR427* locus are averaged over all alignment positions.

B) quantification of all reads that map within *miR427* locus in **A).**

C) Total RNA fragments shorter than 150bp read length over all sequenced ribozero RNA-seq samples. Short RNAs are poorly captured, although there is a peak at length 22bp this corresponds to 58,557 of 738 million total sequenced fragments (0.008%), and only contains two sequences that could be assigned to *miR427*. Therefore, we did not capture mature microRNAs in the ribozero sequencing and the quantification in **A)** corresponds to *pri-miR427*.

Development • Supplementary information

D) Nuclear-cytoplasmic RNA fractionation followed by q-RT-PCR to quantify the levels of *u6* small nuclear RNA and cytoplasmic levels of *odc1* transcripts. *ODC1*-C represents the relative expression levels in cytoplasmic *odc1* expression between the control and *Nup107*-depleted embryos, while *U6*-N shows the comparison of nuclear *u6* levels in the controls and Nup107-depleted embryos. *odc1* signal was not detected in the nuclear fraction, while *u6* expression was not found in the cytoplasm; ns = not statistically significant.

Development • Supplementary information

Table S1. Plasmids used for probe synthesis

Probe Name	Plasmid
foxj1	TNeu058M03
tbxt	TNeu024F07
ectodermin	TNeu104j16
foxI1a	TGas002H16
mixer	TGas105b05
vegT	TGas066f22
gsc	TNeu077f20
vent1	BG487195
chordin	TGas133K5
rest/nrsf	Kindly provided by Alice Paquette (Fox Rothschild LLP)
pri-miR427	IMAGE:7545411 (Owens et al., 2016)
nup107	IMAGE:8850047 (Reza et al., 2016)
nup188	IMAGE:7675066 (Reza et al., 2016)
nup62	IMAGE:7605025 (Reza et al., 2016)
pitx2c	TNeu083k20

Table S2. Primary antibodies used for western blotting

Antibody	Company	Catalog number	Dilution
anti-nup107	Invitrogen	PA5-30774	1:1000
anti-nup188	Bethyl Laboratories	A302-322A	1:1000
mAb414	Abcam	24609	1:1000
anti-GAPDH	Invitrogen	AM4300	1:10,000
anti-β-actin	Santa Cruz	sc-7963	1:10,000
anti-mouse HRP conjugated secondary	Jackson Immuno Research Laboratories	715-035-150	1:10,000
anti-rabbit HRP conjugated secondary	Jackson Immuno Research Laboratories	11-032-171	1:10,000

Development • Supplementary information

References

Owens, N. D. L., Blitz, I. L., Lane, M. A., Patrushev, I., Overton, J. D., Gilchrist, M. J., Cho, K. W. Y. and Khokha, M. K. (2016). Measuring absolute RNA copy numbers at high temporal resolution reveals transcriptome kinetics in development. *Cell Rep,* 14**,** 632-647. doi:10.1016/j.celrep.2015.12.050

Reza, N., Khokha, M. K. and Del Viso, F. (2016). Nucleoporin gene expression in Xenopus tropicalis embryonic development. *Int J Dev Biol,* 60**,** 181-8. doi:10.1387/ijdb.150317nr

Development • Supplementary information
