# Supplementary material for: Nup107 contributes to the maternal-to-zygotic transition by preventing the premature nuclear export of pri-miR427
Source: Development. 2025 Feb 4;152(2):dev202865. doi: 10.1242/dev.202865 (PMC11829755; doi:10.1242/dev.202865)
Supplement: Supplementary file 1 [file develop-152-202865_review_history.pdf]

Nup107 contributes to the maternal to zygotic transition by preventing the premature nuclear export of pri-miR427

Valentyna Kostiuk, Rakib Kabir, Kaitlin Levangie, Stefany Empke, Kimberly Morgan, Nick D.L. Owens, C. Patrick Lusk and Mustafa Khokha
DOI: 10.1242/dev.202865

Editor: Peter Rugg-Gunn

Review timeline

Original submission:	9 March 2024
Editorial decision:	26 April 2024
First revision received:	30 October 2024
Accepted:	18 December 2024

Original submission

First decision letter

MS ID#: DEVELOP/2024/202865

MS TITLE: Nup107 contributes to the maternal to zygotic transition by preventing the premature nuclear export of pri-miRNA 427

AUTHORS: Valentyna Kostiuk, Rakib Kabir, Kimberly Morgan, Nick D.L. Owens, Patrick C. Lusk, and Mustafa Khokha

I have now received all the referees' reports on the above manuscript, and have reached a decision. The referees' comments are appended below, or you can access them online: please go to BenchPress and click on the 'Manuscripts with Decisions' queue in the Author Area.

As you will see, the referees express considerable interest in your work, but have some significant criticisms and recommend a substantial revision of your manuscript before we can consider publication. If you are able to revise the manuscript along the lines suggested, which may involve further experiments, I will be happy receive a revised version of the manuscript. Your revised paper will be re-reviewed by one or more of the original referees, and acceptance of your manuscript will depend on your addressing satisfactorily the reviewers' major concerns. Please also note that Development will normally permit only one round of major revision. If it would be helpful, you are welcome to contact us to discuss your revision in greater detail. Please send us a point-by-point response indicating your plans for addressing the referees' comments, and we will look over this and provide further guidance.

Please attend to all of the reviewers' comments and ensure that you clearly highlight all changes made in the revised manuscript. Please avoid using 'Tracked changes' in Word files as these are lost in PDF conversion. I should be grateful if you would also provide a point-by-point response detailing how you have dealt with the points raised by the reviewers in the 'Response to Reviewers' box. If you do not agree with any of their criticisms or suggestions please explain clearly why this is so.

Reviewer 1

Advance summary and potential significance to field

© 2025. Published by The Company of Biologists under the terms of the Creative Commons Attribution License (https://creativecommons.org/licenses/by/4.0/).

The nuclear pore complex (NPC) is a large protein complex consisting of ~30 nucleoporins (Nups). It remains unclear how differential expression of Nups in different cell types and developmental stages contributes to cellular processes. In this manuscript, Kostiuk et al. find that Nup107, one of the scaffold Nups, varies in expression during early embryonic development in Xenopus. The authors show that depletion of Nup107 leads to abnormal germ layer differentiation and changes in the expression of hundreds of genes. Many of the differentially expressed genes are enriched for sites of miR427/430, a key regulator of maternal transcript clearance, and the authors show that mature miR427 is reduced in nup107-depleted embryos. The authors propose that the reduction in mature miR427 levels is not due to a reduction in miR427 transcription, but due to premature export of pri-miR427 from the nucleus. Overall, the data presented are interesting and well explained. However, I have a few concerns that I think should be addressed before publication.

Comments for the author

1. My main concern is the specificity of the proposed mechanisms. Since NPCs could affect many cellular processes, it is unclear to me whether the effect of changes in nup107 is as specific to the miR427 pathway as the authors propose, or if the effect is rather non-specific but miR427 shows up as a top candidate simply because of its high abundance and central role during the early developmental stages.
The authors argue that “the depletion of nup133 and nup160 is distinct from nup107 …” (page 20, line 12-), but I am confused about how the authors draw this conclusion. Fig S6 suggests to me that the phenotype of nup133 depletion is quite overlapping with nup107 depletion (except for mixer). It is known that the depletion of scaffold Nups, including Nup107 and Nup133, impairs NPC assembly and leads to a reduction in the number of NPCs (e.g., Sakuma et al., Cancer Discovery, 2021). It is unclear to me whether the phenotypes in Nup107 embryos can be attributed specifically to Nup107 or are simply due to loss of NPC integrity caused by the depletion of a scaffold Nup.
It appears that the authors assume that total Nup107 levels detected by WB would correspond to the abundance of Nup107 in the NPC, but this may not necessarily be the case given that excess Nup proteins are deposited in annulate lamellae in early Xenopus embryos. The authors may directly visualize Nup107 and other Nups in the nuclear membrane using the antibodies used in the WB (Fig 1) to see if Nup107 is depleted from NPCs during early embryonic development.

2. I am confused about the phenotype of nup107 MO and nup107 CRISPR KO. My understanding is that MO blocks maternal nup107 transcripts while CRISPR KO blocks zygotic transcription of nup107 without affecting maternal nup107 transcripts. Why do they result in a similar phenotype (Fig 2)? The maternal transcript clearance is impaired in MO embryos before widespread ZGA (page 21, line 22-), but would this be normal in KO embryos until ZGA? Or is nup107 an early ZGA gene?

3. The loss of nuclear retention of pri-miR427 in nup107-depleted embryos is intriguing, but it is unclear if this is sufficient to explain the reduction of mature miR427 in nup107-depleted embryos. I understand that Drosha would be nuclear localized, but in the early embryos there is a large pool of “nuclear” proteins that are maternally deposited in the cytoplasm. The authors may inject pri-miR427 and see if pri-miR427 is not processed and thus has no effect on downstream miR427 targets.

4. I do not think that odc1 is a great control since its localization appears to be diffuse even in control embryos. The authors should use other transcripts that are retained in the nucleus in control embryos.
Otherwise, it is unclear to me whether the effect of nup107 depletion on transcripts localization is specific to miR430 (page 27, line 10-).

Additional comments:
- The claim that “NPC is generally thought to be invariant …” may be a bit outdated, as the field is now well aware of the heterogeneity of NPCs (composition (e.g, Ori et al., Molecular Systems Biology, 2013), post-translational modifications, and structural isoforms), although I agree with the authors that it is poorly understood how variations in NPCs affect cell physiology and development.

<u>Reviewer 2</u>

© 2025. Published by The Company of Biologists under the terms of the Creative Commons Attribution License (https://creativecommons.org/licenses/by/4.0/).

Advance summary and potential significance to field

In this interesting manuscript the authors investigate developmental roles for nuclear pore complex components, focusing on a pathway involving nup107, mIR427 and cell fate determination during the maternal-zygotic transition. Their initial observation is that the levels of specific nucleoporins including y complex component nup107 and scaffold ring nup188, change in early development. In constrast to an unvarying FG nup 62, nup 188 levels go up and down, and nup 107 is largely absent after early gastrulation, Stage 10. In fact many nups change their levels in early development (Supp Fig 2). CRISPR or MO disruption of nup 107 gene leads to abnormal expression of tissue specific markers in the gastrula.
Interestingly, ectoderm appears to be strongly expanded, at the cost of mesoderm and endoderm expression, and with accompanying DV patterning issues and gastrulation. To determine how nup 107 is linked to abnormal germ layer patterning, the authors performed RNA-seq on embryos whose Nup 107 expression had been disrupted. They found altered expression of a variety of genes. Elevated levels of maternal RNAs stood out (Cluster 1). As such, they decided to focus on factors that control maternal RNA degradation, including miR427 (miR430 family).
In particular whether nup107 disruption would altered miR-427 regulation and that this would impact cell fate determination during gastrulation. They found processed miR427 levels reduced in the nup107 MO knockdown, and that co-injection of miR427 in nup107 MO embryos could rescue the germ layer patterned defects. They identify the transcription factor 'rest' as likely larget of miR427, and show it is elevated in the nup107 MO embryos. Finally they confirm that nup107 disruption affects the nuclear localization of pri-mir427, and therefore conclude a role for nup107 is to prevent premature export and possibly degradation of this key component.

Strengths of the work include an tour-de-force of experimentation, determination of expression levels of nucleoporins in early development and revelation of a link between nups, miR427, maternal-zygotic transition and germ layer patterning. I am particularly impressed with the number of rescue controls showing that the effects are specific to Nup107, miR427.

Comments for the author

I have a few comments for improving the manuscript, which are summarized below.

1. In normal development, many of Nups are elevated prior to ZGA, and degraded in gastrulation and beyond (Supp Fig 2). What would be the role for this degradation. In some ways, one might expect Nup 107 depletion and premature nuclear exit of pri-mIR427 to actually an early reduction in maternal RNAs. The authors find the opposite, arguing that premature nuclear export leads to reduced levels of pre-miR427 and thus reduced processing and generation of. Can the author postulate a mechanism or cite studies that show loss of nuclear localized pre-miRs is expected to reduce mIRs

2. If the general diffusion barrier is lost in nup107 MO as suggested by the authors in the discussion, does this mean that general NC partitioning is also disrupted. A recent study by Wuhr Lab (Nguyen et al Nature Comm 2022) suggests that NC partition and timed nuclear entry plays a role in ZGA. Further, nuclear retention of cell cycle regulators is critical for fast cell cycle divisions. Can the author comment on the potential for pleiotropic effects on ZGA or maternal RNA degradation based on general loss of NPC function? Have they quantified cleavage stage divisions in blastula and do these embryo show cell cycle defects, such as an extended division period?

3. Rescue via MO knockdown of rest (Fig 6B). This effect is modest and complicated by the fact that depletion of rest on its own leads to abnormal development. Ideally the authors would have focused just on over-expression phenotype for ectoderm markers and shown that rest MO relieves this. However their data presentation in 6B is odd in that it focuses only on normal expression.
I suggest the authors provide a plot with % embryos with elevated ectodermal markers via in situ and/or other method

4. Data reproducibility. The RNAscope data from Fig 7B appears to be show line scans from individual cells. However, it would strengthen the work to show line scan averages from perhaps

© 2025. Published by The Company of Biologists under the terms of the Creative Commons Attribution License (https://creativecommons.org/licenses/by/4.0/).

20-30 cells/nuclei to provide confidence intervals. Surely they have many images of these nuclei. The legend did not indicate that these were averages

5. Expression data for germ layer genes in nup107 MO embryos. The authors show classes of expression, but I could not find plots in the main or supplemental figures that show how expression is altered for ectoderm, mesoderm and endoderm genes specifically from blastula through late gastrula. Given the altered expression from in situ hybridization, one would expression to find similar alterations in the RNA-seq data. However, the authors only present gene clusters in Fig 2. It would improve the manuscript to demonstrate how germ layer expression is disrupted in the RNA-seq d

Reviewer 3

Advance summary and potential significance to field

Review: Kostiuk et al

The manuscript by Kostiuk et al looks at a role for nup107, which is a nuclear pore protein, in the maternal to zygotic RNA transition of early Xenopus embryos. microRNA-427 has previously been shown to be critical for the degradation of maternal transcripts when zygotic transcription starts. The authors suggest that nup107 regulates the levels of active miR427 by preventing the premature export of pri-miR427 before the completion of processing by drosha.

The authors initially show nup107 to be dynamically expressed in the embryos which contradicts the idea that the nuclear pore complex is generally invariant within different cell types. They then go on to show by RNA-seq that depletion of nup427 delays the degradation of maternal transcripts and that the transcripts enriched by the depletion are enriched in miR427 recognition sites. They further show that miR427 is prevented from nuclear export by nup427. Thus, when the levels of nup107 decrease in the late blastula miR427 activity thus increases.

I found this to be an interesting paper providing new insight into how the transition from maternal to zygotic transcription occurs at a critical stage in development. It also adds another level of potential regulation of microRNA activity. It will be interesting to see if this type of regulation occurs elsewhere in development and also if this is specific for certain microRNAs or microRNAs in general. The work nicely complements recent discoveries in zebrafish and hESCs

Comments for the author

Specific comments

At the start of the results the authors state they are ‘surprised by the finding that nup107 protein levels are diminished after gastrulation’. For a start I would say that they are diminished before gastrulation and that supp fig2a also highlights other nups of interest ie nup42 and 54
Supplemental figure 3 could do with representative pictures of the phenotypes being added. When was the phenotype scored? The legend needs more detail. For instance, in the text the number 36% is used for embryos that did not gastrulate properly. How was this number determined? What does abnormal heart looping have to do with gastrulation?
One question not asked – for which I am not expecting an answer - is what regulates the decrease of nup107 during mid-late blastula?

Minor points

Supplemental fig7 some of the panels are mislabelled in the main text

---

First revision

© 2025. Published by The Company of Biologists under the terms of the Creative Commons Attribution License (https://creativecommons.org/licenses/by/4.0/).

Author response to reviewers' comments

We thank the editor and reviewers for their enthusiasm about our work. We appreciate the acknowledgement of the novelty of our work *("determination of expression levels of nucleoporins in early development and revelation of a link between nups, miR427, maternal-zygotic transition and germ layer patterning… providing new insight into how the transition from maternal to zygotic transcription occurs at a critical stage in development. It also adds another level of potential regulation of microRNA activity"). The reviewers also complimented* our meticulous methodology *("tour-de-force of experimentation,… the number of rescue controls showing that the effects are specific to Nup107, miR427").*

We have made multiple changes to the text and added additional data to address Reviewers' concerns. We feel that these changes have substantively improved the manuscript and hope that this paper will be accepted.

Reviewer 1 Comments for the Author:

1. My main concern is the specificity of the proposed mechanisms. Since NPCs could affect many cellular processes, it is unclear to me whether the effect of changes in nup107 is as specific to the miR427 pathway as the authors propose, or if the effect is rather non-specific but miR427 shows up as a top candidate simply because of its high abundance and central role during the early developmental stages. The authors argue that "the depletion of nup133 and nup160 is distinct from nup107 …" (page 20, line 12-), but I am confused about how the authors draw this conclusion. Fig S6 suggests to me that the phenotype of nup133 depletion is quite overlapping with nup107 depletion (except for mixer). It is known that the depletion of scaffold Nups, including Nup107 and Nup133, impairs NPC assembly and leads to a reduction in the number of NPCs (e.g., Sakuma et al., Cancer Discovery, 2021). It is unclear to me whether the phenotypes in Nup107 embryos can be attributed specifically to Nup107 or are simply due to loss of NPC integrity caused by the depletion of a scaffold Nup. It appears that the authors assume that total Nup107 levels detected by WB would correspond to the abundance of Nup107 in the NPC, but this may not necessarily be the case given that excess Nup proteins are deposited in annulate lamellae in early Xenopus embryos. The authors may directly visualize Nup107 and other Nups in the nuclear membrane using the antibodies used in the WB (Fig 1) to see if Nup107 is depleted from NPCs during early embryonic development.

Response: We agree that Nup107 can have multiple functions. However, miR427 is a top candidate based on RNA-Seq results and rescue experiments. If other factors contributed significantly to this phenotype, overexpression of miR427 would not provide rescue of germ layer expression at stage 10. Moreover, we added new data (Supplemental Figure 8) showing the rescue of abnormal *pitx2c* expression at stage 28 in *nup107*-depleted embryos by miR427 overexpression. The *nup107* depletion phenotype appears to be different from the phenotypes we observed after the depletion of other outer ring nucleoporins (in addition to *mixer*, *foxi1a* and *ectodermin* are not elevated and *vegT* is not reduced in *nup133*-depleted embryos).

We acknowledged in the *Discussion* section that while Nup107 depletion might have an effect on other transcripts and a more general effect on the NPC structure, we chose to focus on miR427 since RNA-Seq results showed its abundant expression at early stages associated with the onset of maternal-zygotic transition and its known role in this process. We also agree with the comment regarding the subcellular localization of Nup107 within the NPC. While we attempted to detect the presence of Nup107 in the NPC using both confocal and expansion microscopy, we could not find an antibody with specificity for immunofluorescence staining. Overall, we agree with the Reviewer and have softened any statements suggesting the phenotype is only specific to *miR427*.

"Additionally, we investigated whether the overexpression of miR427 had an impact on events later in embryonic development by examining the expression of pitx2c, a marker of Left-Right (LR) patterning. While 28% of nup107-depleted embryos had abnormal pitx2c expression, the concurrent overexpression of miR427 in these embryos resulted in 11% of abnormal pitx2c phenotypes (Supplemental Figure 8). This suggests that in addition to its crucial role in maternal RNA clearance during early development, abnormal levels of miR427 affect multiple downstream events, including LR patterning."

2.I am confused about the phenotype of nup107 MO and nup107 CRISPR KO. My understanding is that MO blocks maternal nup107 transcripts while CRISPR KO blocks zygotic transcription of nup107 without affecting maternal nup107 transcripts. Why do they result in a similar phenotype (Fig 2)? The maternal transcript clearance is impaired

© 2025. Published by The Company of Biologists under the terms of the Creative Commons Attribution License (https://creativecommons.org/licenses/by/4.0/).

in MO embryos before widespread ZGA (page 21, line 22-), but would this be normal in KO embryos until ZGA? Or is nup107 an early ZGA gene?

Response: The reviewer is correct that one might expect that Nup107 depletion by MO or CRISPR might give different phenotypes because of inhibition of maternal vs zygotic transcripts. However, in this case we got similar phenotypes with both depletion strategies suggesting that the zygotic transcripts are most relevant in generating the depletion phenotype. It also reflects that our depletion strategies are specific to Nup107, which is also evident in our rescue experiments with human Nup107 transcripts.

3. The loss of nuclear retention of pri-miR427 in nup107-depleted embryos is intriguing, but it is unclear if this is sufficient to explain the reduction of mature miR427 in nup107- depleted embryos. I understand that Drosha would be nuclear localized, but in the early embryos there is a large pool of "nuclear" proteins that are maternally deposited in the cytoplasm. The authors may inject pri-miR427 and see if pri-miR427 is not processed and thus has no effect on downstream miR427 targets.

Response: We contemplated these experiments as well. However, the pri-miR427 is 55kb long and is too large to be synthesized. Shorter fragments of pri-miR427 do not have the same structural confirmation to be recognized and cleaved by Drosha as the full pri-miR427 transcript.(Ruiz-Arroyo & Nam, 2022) We also thought about using pre-miR427, which is 64bp long. However, this intermediate structure has a transient function in the miRNA processing cascade and gets processed and degraded relatively quickly. While we attempted to detect this intermediate structure with both Northern blot and q-RT-PCR, our attempts were unsuccessful. In addition, we tried to detect Drosha subcellular localization using immunofluorescence, but we could not detect a specific immunofluorescence signal with the Drosha antibody (previously shown to work by Western blot in *Xenopus*).(Muggenhumer et al., 2014)

4. I do not think that odc1 is a great control since its localization appears to be diffuse even in control embryos. The authors should use other transcripts that are retained in the nucleus in control embryos. Otherwise, it is unclear to me whether the effect of nup107 depletion on transcripts localization is specific to miR430 (page 27, line 10-).

Response: We performed additional nuclear-cytoplasmic RNA fractionation followed by q- RT-PCR experiments to quantify the levels of U6 small nuclear RNA and cytoplasmic levels of ODC1 transcript. We detected ODC1 transcripts in the cytoplasm only ("ODC1-C"), while U6 transcripts localized only to the nucleus ("U6-N") (even in *nup107*-depleted embryos).
When comparing the differences in signal levels between UIC and Nup107-depleted embryos in the cytoplasm and nucleus for ODC1 and U6, respectively, we found no significant differences. These results showed that Nup107 depletion did not affect subcellular localization of ODC1 or U6 transcripts, further supporting its specific effect on pri-miR427 localization. These data have been added as Supplemental Figure 9D.

Figures

Supplemental Figure 9D

D) Nuclear-cytoplasmic RNA fractionation followed by q-RT-PCR to quantify the levels of *U6* small nuclear RNA and cytoplasmic levels of *ODC1* transcripts. *ODC1*-C represents the relative expression levels in cytoplasmic *ODC1* expression between the control and *Nup107*-depleted embryos, while *U6*-N shows the comparison of nuclear *U6* levels in the controls and *Nup107*-depleted embryos. *ODC1* signal was not detected in the nuclear fraction, while *U6* expression was not found in the cytoplasm.

© 2025. Published by The Company of Biologists under the terms of the Creative Commons Attribution License (https://creativecommons.org/licenses/by/4.0/).

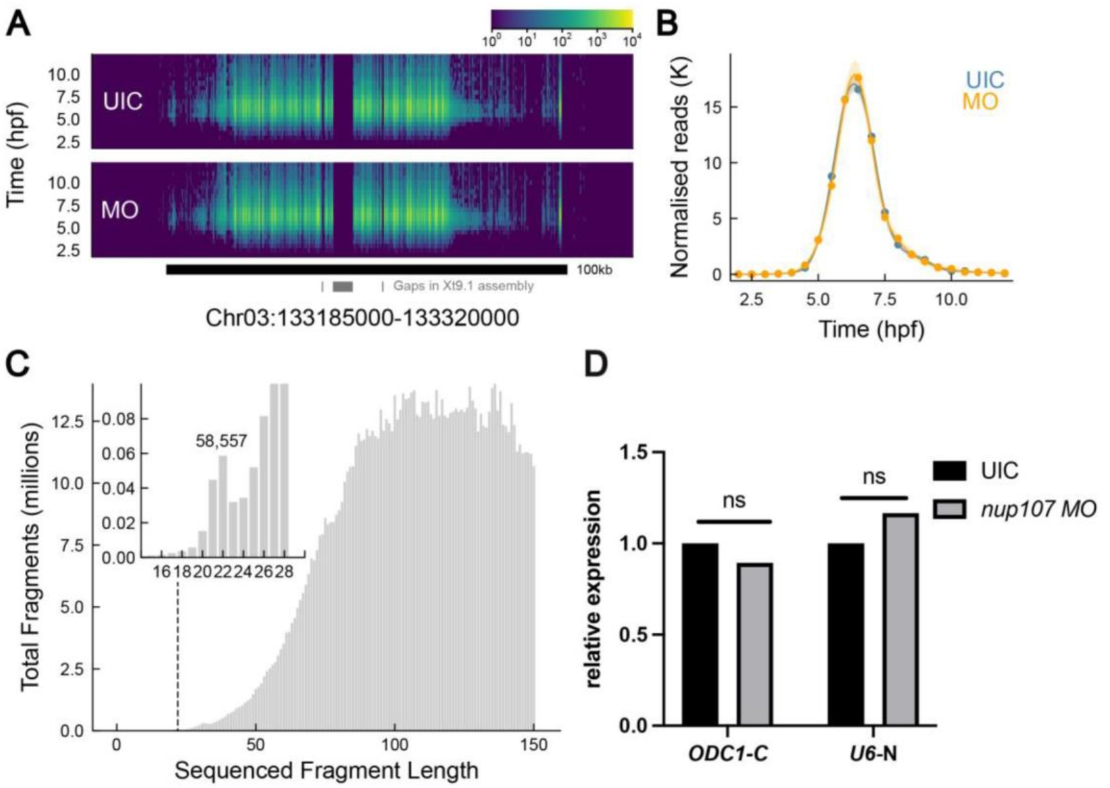

Results

“To test whether this is specific to *miR427*, we examined the subcellular localization of other transcripts in *nup107*-depleted embryos. Following nuclear-cytoplasmic fractionation and RNA extraction, we quantified the levels of *ODC1* and *U6*, known cytoplasmic and nuclear transcripts, respectively. In both control and *nup107*-depleted embryos, we identified comparable levels of ODC1 in the cytoplasmic fraction and U6 in the nuclear fraction only (Supplemental Figure 9D). This result supports the notion that *nup107* depletion does not have a broad effect on other nuclear localized transcripts.”

Acknowledgements

“We thank Mason McCool for assistance with subcellular fractionation.”

Additional comments:
- The claim that “NPC is generally thought to be invariant …” may be a bit outdated, as the field is now well aware of the heterogeneity of NPCs (composition (e.g, Ori et al., Molecular Systems Biology, 2013), post-translational modifications, and structural isoforms), although I agree with the authors that it is poorly understood how variations in NPCs affect cell physiology and development.

Response: We changed the introduction statement in the *Abstract* to emphasize this important point.

“Emerging evidence suggests that the nuclear pore complex can have unique compositions and distinct nucleoporin functions in different cells.”

Reviewer 2 Comments for the Author:
I have a few comments for improving the manuscript, which are summarized below.

1. In normal development, many of Nups are elevated prior to ZGA, and degraded in gastrulation and beyond (Supp Fig 2). What would be the role for this degradation. In some ways, one might expect Nup 107 depletion and premature nuclear exit of pri-mIR427 to actually an early reduction in maternal RNAs. The authors find the opposite, arguing that premature nuclear export leads to reduced levels of pre-miR427 and thus reduced processing and generation

© 2025. Published by The Company of Biologists under the terms of the Creative Commons Attribution License (https://creativecommons.org/licenses/by/4.0/).

of. Can the author postulate a mechanism or cite studies that show loss of nuclear localized pre-miRs is expected to reduce mIRs?

Response: We did not find any studies demonstrating a similar mechanism of primary miRNA retention in the nucleus by nucleoporins. While other papers demonstrated the role of Nup107 in mRNA export, to our knowledge, the current study is the first one to show that *nup107* depletion leads to the cytoplasmic localization of uncleaved pri-miR427 transcript with subsequent reduction in mature miR427 levels. With regard to mechanism, we propose that premature export of pri-miR427 prevents its cleavage by Drosha, which is nuclear localized.

2. If the general diffusion barrier is lost in nup107 MO as suggested by the authors in the discussion, does this mean that general NC partitioning is also disrupted. A recent study by Wuhr Lab (Nguyen et al Nature Comm 2022) suggests that NC partition and timed nuclear entry plays a role in ZGA. Further, nuclear retention of cell cycle regulators is critical for fast cell cycle divisions. Can the author comment on the potential for pleiotropic effects on ZGA or maternal RNA degradation based on general loss of NPC function? Have they quantified cleavage stage divisions in blastula and do these embryo show cell cycle defects, such as an extended division period?

Response: We did not observe any gross cleavage defects or delays in early development between the control and *nup107*-depleted embryos. Additionally, based on the RNA-Seq quantification of pri-miR427 levels and timing of transcription (Supplemental Figure 9B), the timing of ZGA is not changed between the control and *nup107*-depleted embryos. Instead, we see a delayed maternal transcript clearance. Therefore, we concluded that the *nup107* depletion primarily affected the clearance of maternal RNAs during the ZGA process rather than its initiation in contrast to what was described by Wuhr and colleagues.

3. Rescue via MO knockdown of rest (Fig 6B). This effect is modest and complicated by the fact that depletion of rest on its own leads to abnormal development. Ideally the authors would have focused just on over-expression phenotype for ectoderm markers and shown that rest MO relieves this. However, their data presentation in 6B is odd in that it focuses only on normal expression. I suggest the authors provide a plot with % embryos with elevated ectodermal markers via in situ and/or other method

Response: First, when we deplete *rest,* we get a decrease in *ectodermin* and *foxi1a* expression, and the opposite is true when we deplete *nup107*. To demonstrate that the depletion of *rest* rescues *nup107* depletion phenotype, we added new data describing normal, increased and decreased expression of ectodermal markers per the reviewer's suggestion. In this way, each of the categories of gene expression levels are represented in the new graphs (Figure 6B). The data support that depletion of *rest* rescues the *nup107* depletion phenotype consistent with our model that *nup107* suppresses *rest* transcript levels. We updated Figure 6B to show these data.

© 2025. Published by The Company of Biologists under the terms of the Creative Commons Attribution License (https://creativecommons.org/licenses/by/4.0/).

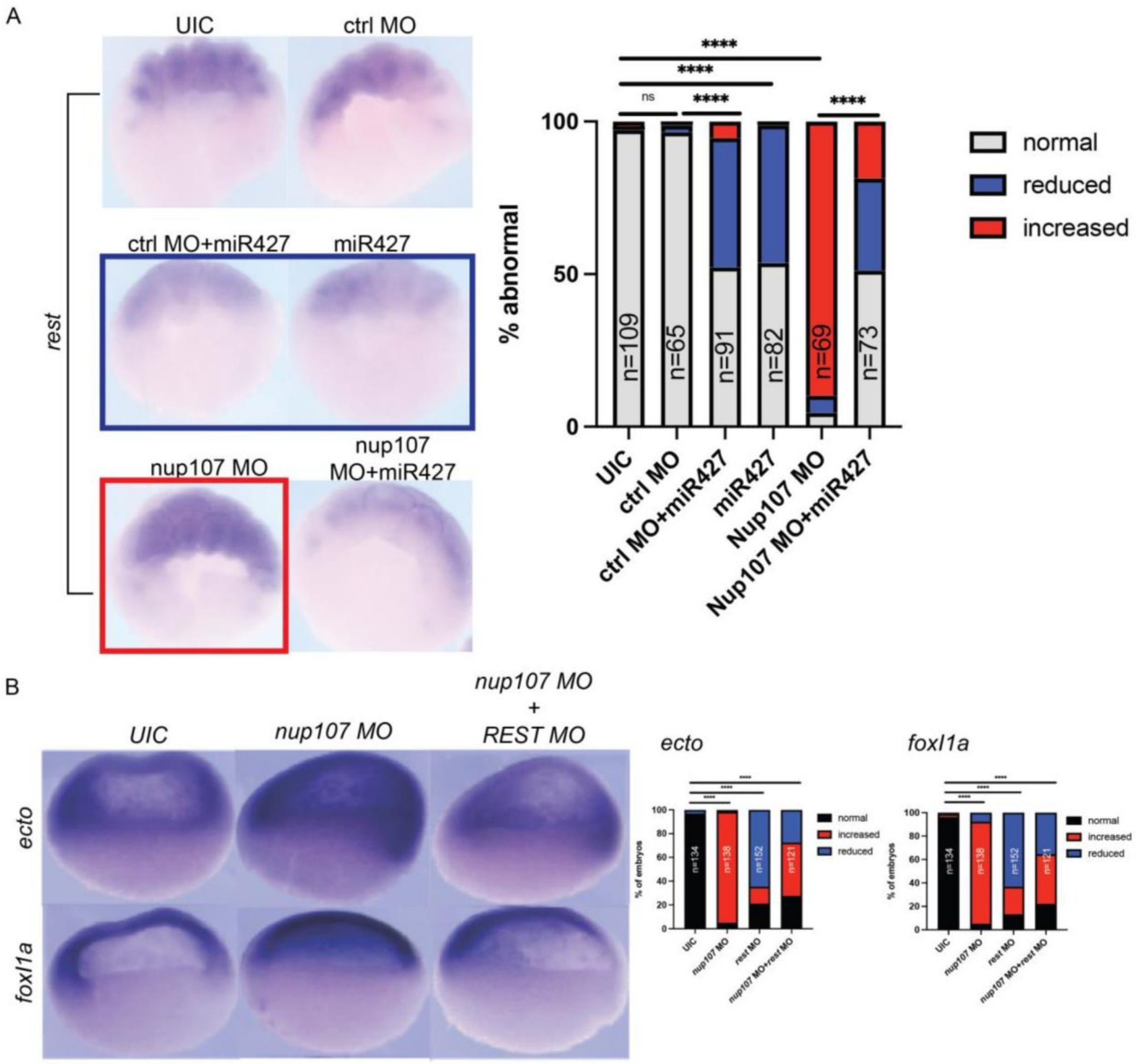

4. Data reproducibility. The RNAscope data from Fig 7B appears to be show line scans from individual cells. However, it would strengthen the work to show line scan averages from perhaps 20-30 cells/nuclei to provide confidence intervals. Surely they have many images of these nuclei. The legend did not indicate that these were averages.

Response: We added this information to the figure legend demonstrating the reproducibility.

“The following number of embryos were used for this experiment: sense pri-miR427 probe: 16 *nup107*-depleted embryos and 20 control embryos; antisense pri-miR427 probe: 20 *nup107*-depleted embryos and 18 control embryos; antisense ODC1 probe: 19 *nup107*- depleted embryos and 17 control embryos. The confidence intervals for measurements were: sense pri-miR427 probe: *nup107* MO sample 1.3±0.6 [0.7–1.8], UIC sample 0.9±0.4 [0.6–1.3]; antisense pri-miR427 probe: nup107 sample 10.3±3.4 [6.9–13.8], UIC sample 18.8±7.4 [11.4-26.2]; antisense ODC1 probe: UIC sample 5.7±0.8 [4.9– 6.5], nup107 MO sample 6.3±0.8 [5.5–
7.0].”

5. Expression data for germ layer genes in nup107 MO embryos. The authors show classes of expression, but I could not find plots in the main or supplemental figures that show how expression is altered for ectoderm, mesoderm and endoderm genes specifically from blastula through late gastrula. Given the altered expression from in situ hybridization, one would expression to find similar alterations in the RNA-seq data. However, the authors only present gene clusters in Fig 2. It would improve the manuscript to demonstrate how germ layer expression is disrupted in the RNA-seq

© 2025. Published by The Company of Biologists under the terms of the Creative Commons Attribution License (https://creativecommons.org/licenses/by/4.0/).

Response: While we detected trends of germ layer gene expression similar to the *in situ* results, the differences between control and *nup107*-depleted embryos were not statistically significant and therefore not presented in the paper. This could be explained by the fact that the RNA-Seq experiment was performed using pools of multiple whole embryos, while *in situ* hybridization experiments allowed for a better resolution of spatial signals at the level of tissue in an individual embryo. Additionally, we included two papers in the *Discussion* section describing phenotypes consistent with our results of germ layer and dorsal Organizer patterning changes in the setting of targeted depletion of miR427 in *Xenopus laevis* and human embryonic stem cells (ESCs) (Rosa et al., 2009) and genetic deletion of miR430 cluster in zebrafish.(Liu et al., 2020)

Reviewer 3 Comments for the Author:

Specific comments

1. At the start of the results the authors state they are 'surprised by the finding that nup107 protein levels are diminished after gastrulation'. For a start I would say that they are diminished before gastrulation and that supp fig2a also highlights other nups of interest ie nup42 and 54.

Response: Thank you for this comment. We updated this statement in the text:

"Surprised by the finding that *nup107* protein levels are diminished **prior to gastrulation**, we investigated a role for *nup107* at this timepoint. We used CRISPR/Cas9 to introduce indels into the Nup107 gene to ablate its expression."

2. Supplemental figure 3 could do with representative pictures of the phenotypes being added. When was the phenotype scored? The legend needs more detail. For instance, in the text the number 36% is used for embryos that did not gastrulate properly. How was this number determined? What does abnormal heart looping have to do with gastrulation?

Response: Thank you, we regret that we did not clarify this previously. We added images of abnormal gastrulation in these embryos. Embryos were divided into "normal" and "abnormal" groups at stage 15 based on the complete or incomplete closure of the blastopore lip, respectively (***updated*** **Supplemental Figure 3 and Figure Legend).** Proper heart looping occurs following the formation of the Left-Right Organizer, which is a derivative of the superficial dorsal mesoderm formed at the completion of gastrulation. From these data, we concluded that abnormal cardiac looping was associated with abnormal gastrulation in *nup107*-depleted embryos.

"Supplemental Figure 3. The comparison of abnormal heart looping in embryos with normal (complete closure of the blastopore lip, black arrow) and abnormal gastrulation (incomplete closure of the blastopore lip, red arrow)."

© 2025. Published by The Company of Biologists under the terms of the Creative Commons Attribution License (https://creativecommons.org/licenses/by/4.0/).

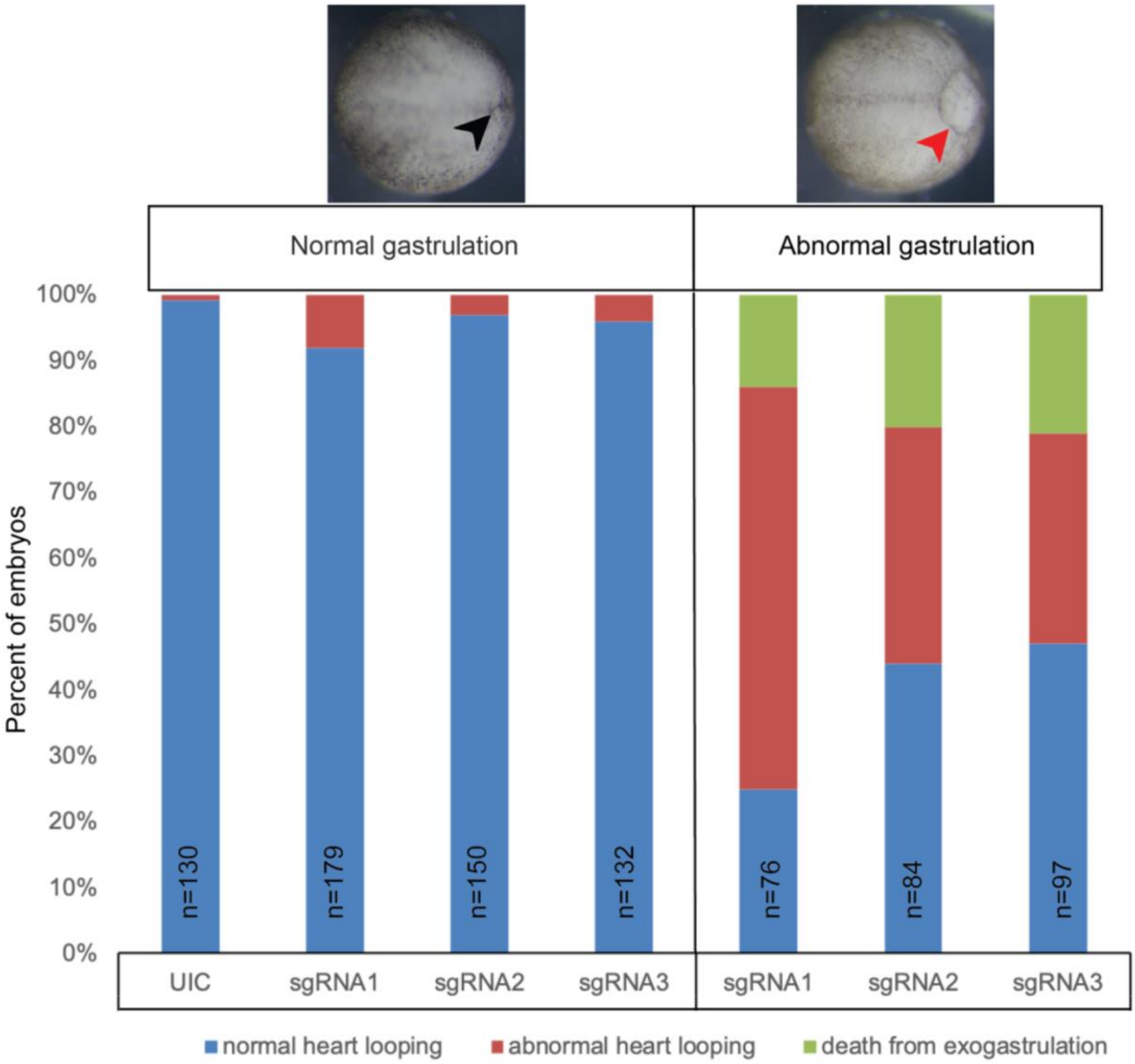

3. One question not asked - for which I am not expecting an answer - is what regulates the decrease of nup107 during mid-late blastula?

Response: Thank you for this interesting question. We do not know if this process involves active protein degradation or a failure of protein production. We do intend to investigate this in the future.

Minor points

4. Supplemental fig7 some of the panels are mislabelled in the main text

Response: Thank you for pointing it out. We corrected this.

References:

Liu, Y., Zhu, Z., Ho, I. H. T., Shi, Y., Li, J., Wang, X.,…Cheng, C. H. K. (2020). Genetic Deletion of. *Front Genet*, *11*, 853. https://doi.org/10.3389/fgene.2020.00853
Muggenhumer, D., Vesely, C., Nimpf, S., Tian, N., Yongfeng, J., & Jantsch, M. F. (2014). Drosha protein levels are translationally regulated during Xenopus oocyte maturation. *Mol Biol Cell*, *25*(13), 2094-2104. https://doi.org/10.1091/mbc.E13-07-0386
Ruiz-Arroyo, V. M., & Nam, Y. (2022). Dynamic Protein-RNA recognition in primary MicroRNA processing. *Curr Opin Struct Biol*, *76*, 102442. https://doi.org/10.1016/j.sbi.2022.102442

© 2025. Published by The Company of Biologists under the terms of the Creative Commons Attribution License (https://creativecommons.org/licenses/by/4.0/).

Second decision letter

MS ID#: DEVELOP/2024/202865

MS TITLE: Nup107 contributes to the maternal to zygotic transition by preventing the premature nuclear export of pri-miRNA 427

AUTHORS: Valentyna Kostiuk, Rakib Kabir, Kaitlin Levangie, Stefany Empke, Kimberly Morgan, Nick D.L. Owens, Patrick C. Lusk, and Mustafa Khokha

I am happy to tell you that your manuscript has been accepted for publication in Development, pending our standard publication integrity checks.

Reviewer 1

Advance summary and potential significance to field

The nuclear pore complex (NPC) is a large protein complex consisting of ~30 nucleoporins (Nups). It is unclear how differential expression of Nups in different cell types and developmental stages contributes to cellular processes. Kostiuk et al. find that Nup107, one of the scaffold Nups, varies in expression during early embryonic development in Xenopus. The authors show that depletion of Nup107 leads to abnormal germ layer differentiation and changes in the expression of hundreds of genes. Many of the differentially expressed genes are enriched for sites of miR427/430, a key regulator of maternal transcript clearance, and the authors show that mature miR427 is reduced in nup107-depleted embryos. The authors propose that the reduction in mature miR427 levels is not due to a reduction in miR427 transcription, but due to premature export of pri-miR427 from the nucleus. Taken together, this work provides new insights into the emerging role of NPCs in development.

Comments for the author

The authors have addressed my comments in the revised manuscript. I have no additional major comments. Congratulations to the authors for their interesting findings.

Reviewer 2

Advance summary and potential significance to field

The authors reveal roles for nuclear pore complex components including nup107 in mIR427 partitioning and processing significant to maternal RNA degradation and cell fate determination during the maternal-zygotic transition. They found CRISPR or MO disruption of nup 107 gene causes abnormal expression of tissue specific markers in the gastrula. Ectoderm expands, at the cost of mesoderm and endoderm expression, with accompanying DV patterning issues and gastrulation defects. Gene profiling revealed elevated levels of maternal RNAs stand out, implicating a controller of maternal RNA degradation such as miR427. They found processed miR427 levels are reduced in the nup107 MO knockdown, and co-injection of miR427 in nup107 morphant embryos rescue the germ layer patterned defects. This study represents work a tour-de-force of experimentation and reveals a novel regulatory role for Nucleoporins essential for embryo development

Comments for the author

This interesting study has been further strengthened with the revised manuscript. I feel that the authors have satisfied my concerns, which were few, and that the work is ready for publication

© 2025. Published by The Company of Biologists under the terms of the Creative Commons Attribution License (https://creativecommons.org/licenses/by/4.0/).

Reviewer 3

Advance summary and potential significance to field

This is a revised manuscript. The authors have addressed my previous comments. The paper is improved from the first version and now suitable for publication.

Comments for the author

Thank you for responding to my comments.

© 2025. Published by The Company of Biologists under the terms of the Creative Commons Attribution License (https://creativecommons.org/licenses/by/4.0/).
